# Arginine demethylation is catalysed by a subset of JmjC histone lysine demethylases

**DOI:** 10.1038/ncomms11974

**Published:** 2016-06-23

**Authors:** Louise J. Walport, Richard J. Hopkinson, Rasheduzzaman Chowdhury, Rachel Schiller, Wei Ge, Akane Kawamura, Christopher J. Schofield

**Affiliations:** 1 Department of Chemistry, Chemistry Research Laboratory, University of Oxford, Mansfield Road, Oxford OX1 3TA, UK; 2 Division of Cardiovascular Medicine, Radcliffe Department of Medicine, Wellcome Trust Centre for Human Genetics, Roosevelt Drive, Oxford OX3 7BN, UK

## Abstract

While the oxygen-dependent reversal of lysine *N*^ɛ^-methylation is well established, the existence of bona fide *N*^ω^-methylarginine demethylases (RDMs) is controversial. Lysine demethylation, as catalysed by two families of lysine demethylases (the flavin-dependent KDM1 enzymes and the 2-oxoglutarate- and oxygen-dependent JmjC KDMs, respectively), proceeds via oxidation of the *N*-methyl group, resulting in the release of formaldehyde. Here we report detailed biochemical studies clearly demonstrating that, in purified form, a subset of JmjC KDMs can also act as RDMs, both on histone and non-histone fragments, resulting in formaldehyde release. RDM catalysis is studied using peptides of wild-type sequences known to be arginine-methylated and sequences in which the KDM's methylated target lysine is substituted for a methylated arginine. Notably, the preferred sequence requirements for KDM and RDM activity vary even with the same JmjC enzymes. The demonstration of RDM activity by isolated JmjC enzymes will stimulate efforts to detect biologically relevant RDM activity.

## Abstract (web summary)

While reversal of lysine methylation on histone tails is a well-established mechanism to tune gene expression, the existence of a similar arginine demethylation process is controversial. Here, the authors show that some jumonji enzymes possess both lysine and arginine demethylase activity *in vitro*.

## Graphical abstract

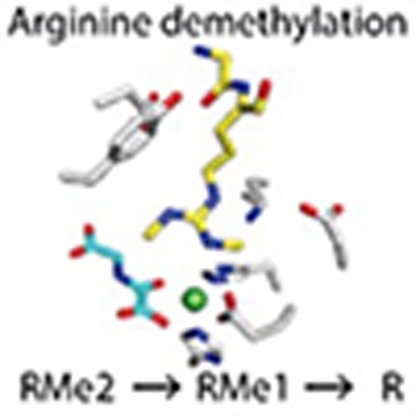

Post-translational modifications, which vastly increase the size of the functional proteome, play central roles in numerous cellular processes, including enzyme catalysis, protein–protein interactions and gene regulation^1^. Modifications to histones, which are especially prevalent on the *N*-terminal tail of histone H3, include *N*^ɛ^-acetylation and ubiquitination of lysine residues, phosphorylation of serine and threonine residues, and *N*^ɛ/ω^-methylation of lysine and arginine residues^2^. Coupled with other factors, these modifications regulate the sets of genes that are transcribed in a time- and context-dependent manner. While lysine acetylation is usually transcriptionally activating, arginine- and lysine methylation can be either activating or repressive, depending on the site and extent of methylation.

Arginine/lysine methylation is catalysed by *S*-adenosyl-methionine-dependent methyltransferases (protein arginine methyltransferases, PRMTs, and lysine methyltransferases, KMTs, respectively)^3^. Although for many years *N*-methylation was thought to be a stable, irreversible modification, the reversal of lysine methylation is now well established. Two families of histone *N*^ɛ^-methyllysine demethylases (KDMs) have been identified; the flavin-dependent KDM1s (lysine-specific demethylase) and the Fe(II)- and 2-oxoglutarate (2OG)-dependent JmjC-domain-containing enzymes. Both families of KDMs operate via methyl-group oxidizing mechanisms that result in the co-production of formaldehyde^4^. Lysine methylation/demethylation can thus occur on a timescale of only two enzyme-catalysed steps, that is, has the potential to be highly dynamic.

In contrast to lysine methylation, the extent to which arginine methylation is dynamic is much less clear. Arginine residues undergo PRMT-catalysed methylation on their terminal guanidine nitrogens to give monomethyl (MMA/Rme), and symmetric (SDMA/Rme2s) and asymmetric (ADMA/Rme2a) dimethylated forms^5^. The peptidyl-arginine deiminases (PADs) catalyse the hydrolysis of the guanidino arginine side chain to the urea group of citrulline. Both PAD2 and PAD4 catalyse citrulline formation at multiple positions on histone tails^6^. In the case of PAD4, this activity has also been reported on monomethylarginine residues^7^, although evidence that this activity occurs in an *in vivo* setting remains limited^8^. In either case, demethylimination is not a ‘true' demethylation reaction—it removes but does not reverse *N*^ω^-methylation. Neutral citrulline is produced, which has considerably different chemical properties compared to unmethylated arginine. One 2OG oxygenase, JMJD6, has been reported to catalyse methylarginine demethylation of histone H3/H4 residues^9^. Some subsequent reports have not reproduced these observations, and JMJD6 has also been assigned as a lysine C-5 hydroxylase acting on mRNA splicing-regulatory proteins, and potentially histone proteins^10^,^11^,^12^. Other recent reports have identified further arginine demethylation activity by JMJD6 on histone and non-histone substrates^13^,^14^,^15^,^16^; however, the biochemical evidence for methylarginine demethylation by JMJD6 is not unequivocal^17^.

Despite the controversial history as potential methylarginine demethylases (RDMs), the 2OG oxygenases represent good candidates for such reactions because of the broad spectrum of oxidative reactions that they catalyse. Some JmjC oxygenases catalyse hydroxylation of lysine residues to give stable hydroxylated products^18^. Arginine demethylation could occur via oxidation of the arginine *N*^ω^-methyl group and concomitant loss of formaldehyde in a reaction analogous to that of lysine demethylation (Fig. 1a,b). In a recent study we found that the JmjC KDMs can catalyse oxidation of *N*^ɛ^-alkyl groups other than methyl groups, including oxidation reactions on isopropyl groups at a position equivalent to that required for oxidation of a *N*^ω^-methylarginine group (Fig. 1c)^19^. Here we report systematic studies on the potential of the catalytic domains of representative human JmjC enzymes to catalyse *N*^ω^-methylarginine demethylation. The results clearly demonstrate that some, but not all, purified JmjC KDMs can act as RDMs.

## Results

### JmjC enzymes have RDM activity

**Figure 2:**
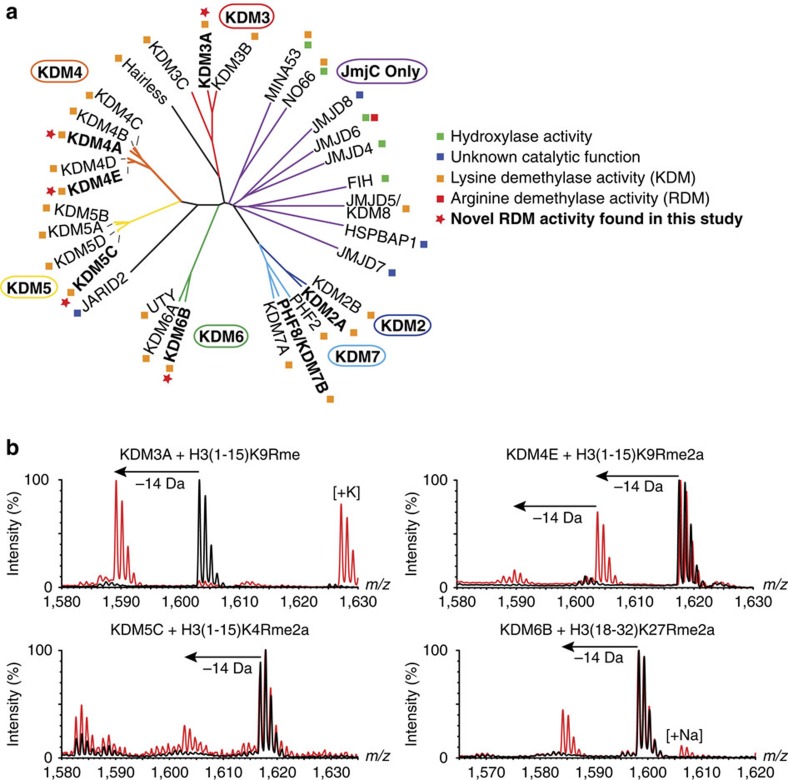
Some JmjC KDMs catalyse arginine demethylation. (**a**) Human JmjC oxygenases grouped according to the sequence analysis of their catalytic domains showing their assigned/proposed functions and novel functions found in this study (red stars). Some assignments are controversial. Proteins used in this study are in bold. KDM4E is a likely pseudogene. (**b**) MALDI-TOF MS analysis of demethylation of arginine-methylated variant histone peptides by truncated recombinant catalytic domain constructs of KDM3A, KDM4E, KDM5C and KDM6B. Red spectra show reactions including enzyme and black spectra the no enzyme control.

To test whether the catalytic domains of the JmjC KDMs can act as RDMs, we produced truncated recombinant proteins containing the catalytic domains of representatives of the six identified human JmjC KDM subfamilies (KDM2A, KDM3A, KDM4E, KDM5C, KDM6B and PHF8 (also known as KDM7B), Fig. 2a)^20^,^21^. The representative JmjC KDMs were tested for *in vitro* demethylation activity with synthetic fragment sequences of the histone H3 tail with mono- and asymmetric/symmetric dimethylated arginine residues replacing the *N*^ɛ^-methylated lysine residues known to be demethylated in established substrates for each JmjC KDM (Supplementary Table 1). The corresponding ‘wild-type' peptides with the naturally positioned *N*^ɛ^-methylated lysine residues were used as positive controls. Reactions were initially analysed using MALDI-TOF mass spectrometry (MS) to test for demethylation (observed as a −14 Da mass shift).

**Figure 3:**
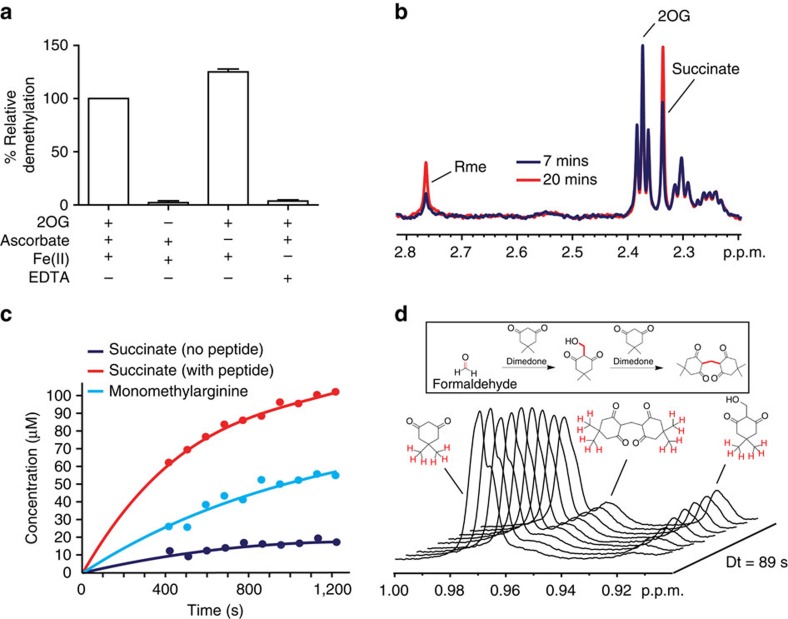
The mechanisms of arginine and lysine demethylation are similar. (**a**) The arginine demethylation reaction of KDM6B requires both 2OG and Fe(II) for active demethylation. The degree of arginine demethylation of H3(14–34)K27Rme2a catalysed by KDM6B was quantified by MALDI-TOF mass spectrometry. Data show the mean±s.e.m. (**b**) ^1^H NMR analyses of KDM6B-catalysed arginine demethylation. The ^1^H spectra are of a reaction mixture containing KDM6B (9 μM), H3(14–34)K27Rme2a peptide (1 mM), 2OG (500 μM), ascorbate (1 mM) and iron(II) (100 μM) after 7 min (blue) and 20 min (red) at 298 K. The resonance corresponding to the methyl group of monomethylated arginine (Rme) is highlighted. (**c**) Graphs showing the degree of succinate production and peptide demethylation of H3(14–34)K27Rme2a catalysed by KDM6B as quantified by ^1^H NMR (700 MHz). (**d**) Detection of formaldehyde release during KDM6B-catalysed arginine demethylation. Dimedone reacts with formaldehyde in aqueous solution to form stable adducts that are detectable using ^1^H NMR (insert, the formaldehyde-derived carbons in the adducts are highlighted red). Incubation of a reaction mixture containing KDM6B (6.5 μM), H3(18–30)K27Rme2a peptide (1 mM), 2OG (4 mM), ascorbate (1 mM) and iron(II) (100 μM) and dimedone (667 μM) revealed the formation of two dimedone adducts using ^1^H NMR (700 MHz). Protons responsible for each peak are shown in red.

For KDM2A and PHF8, demethylation was observed only for the characterized lysine substrates (Supplementary Figs 1 and 2), that is, no RDM activity was detected. Interestingly, however, in addition to demethylation of the characterized lysine substrates, evidence for demethylation of both asymmetric and symmetric dimethyl- and monomethylarginine residues was accrued by MS for KDM3A, KDM4E, KDM5C and KDM6B (Fig. 2b and Supplementary Figs 3–6), that is, product peptide spectra manifested mass shifts of −14 Da (and in some cases −28 Da, corresponding to the loss of two methyl groups) relative to the methylated substrate residue. Under our incubation conditions, such mass shifts were only observed in the mass spectra of reactions in the presence of enzyme. KDM3A, KDM4E and KDM5C catalysed demethylation of both symmetric and asymmetric dimethylarginine residues to give a mixture of both the monomethylated and unmethylated arginine residues. At least under the tested conditions, however, we observed that KDM6B only catalyses a single demethylation of asymmetric dimethylarginine to the monomethylated species. Within our limits of detection, no activity was observed for KDM6B and the tested peptides containing monomethylarginine or symmetric dimethylarginine (Supplementary Fig. 6a). Interestingly, when the KDM6B substrate was extended from 15 to 21 residues, a double demethylation reaction to the unmethylated arginine residue was observed in the MALDI MS of reactions containing KDM6B (Supplementary Fig. 6b). As expected for a 2OG oxygenase-catalysed reaction, demethylation by KDM6B required the presence of the Fe(II) cofactor and 2OG co-substrate (Fig. 3a).

In order to validate the MS-based results, we used NMR spectroscopy to follow KDM6B-catalysed demethylation of the 15- and 21-residue H3K27Rme2a peptides. Immediately after initiation of the reaction, the samples containing KDM6B, with either the 15- or 21-residue peptide substrate and cofactors/co-substrates, were transferred to NMR tubes and then subjected to ^1^H NMR (700 MHz) analyses (Fig. 3b and Supplementary Fig. 7a). In both samples, conversion of 2OG to succinate was evidenced by reduction of the ^1^H resonance at δ_H_ 2.4 p.p.m. (corresponding to the protons attached to C4 of 2OG) with concomitant emergence of a ^1^H resonance at δ_H_ 2.3 p.p.m. (corresponding to the methylene protons of succinate). An additional singlet resonance at δ_H_ 2.8 p.p.m. was also observed to increase in intensity during the analyses; this was assigned to the *N*-methyl protons of the monomethylarginine product based on ^1^H and ^13^C chemical shift analyses (δ_C_ 27.5 p.p.m., as determined by ^1^H-^13^C-HSQC analysis of the sample with the 15-residue peptide, Fig. 3b and Supplementary Fig. 7b). Quantitative analyses on the sample with the 21-residue peptide revealed a faster rate of succinate production relative to the rate of peptide demethylation, indicating some uncoupling of 2OG and peptide oxidation (that is, some conversion of 2OG to succinate without concomitant oxidation of the peptide methyl group). The degree of 2OG oxidation was greater in the presence of peptide than without peptide, suggesting that binding of the peptide to KDM6B stimulates 2OG oxidation but does not always necessarily lead to methyl group oxidation (Fig. 3c and Supplementary Fig. 7c). This ‘uncoupling' of 2OG oxidation and peptide demethylation is well precedented for 2OG oxygenases, although it is less pronounced during KDM4E-catalysed demethylation of methyllysine peptides^22^. Addition of the formaldehyde-trapping reagent dimedone to the NMR reaction mixture in the presence of the 15 residue peptide resulted in the emergence of new ^1^H resonances, which were assigned to two dimedone–formaldehyde adducts (Fig. 3d). These adducts have been previously observed to form upon incubation of dimedone with formaldehyde released during JmjC KDM-catalysed demethylation, and therefore imply that KDM6B-catalysed methylarginine demethylation follows a similar mechanism to that for methyllysine demethylation (that is, methyl group oxidation to release formaldehyde)^22^. The combined MS and NMR results thus reveal that some JmjC oxygenases can catalyse *N*^ω^-methyl arginine demethylation.

Preliminary kinetic parameters for selected methylarginine variant peptides were determined using a formaldehyde dehydrogenase-coupled demethylation assay (Table 1)^23^. In the case of KDM6B, the methylated arginine peptide probably binds more weakly (as judged by *K*_M_ values) in the active site than its methylated lysine analogue (*K*_M_ 676.3±118.3 versus 6.7±0.6 μM). Interestingly, however, the *k*_cat_ value for the arginine peptide was higher than that for the analogous lysine peptide (*k*_cat_ 28.8 × 10^−3^±3.6 × 10^−3^ versus 11.7 × 10^−3^±0.1 × 10^−3 ^s^−1^), suggesting that in the context of a tighter binding substrate (for example, a protein rather than the peptides tested here) it is possible that arginine demethylation by KDM6B is as efficient as, or favoured relative to, lysine demethylation. For KDM5C, the rate of asymmetric dimethylarginine demethylation was also found to be of similar levels to that of lysine demethylation (*k*_cat_ 41.2±3.5 versus 67.1±10.3 s^−1^), while for KDM3A and KDM4E both the rate of demethylation and binding constants were found to be considerably lower for the arginine peptides than the analogous lysine peptides (Table 1). KDM4E appears to bind to the peptide containing symmetric arginine less well than the asymmetric residue (*K*_M_ 101.1±11.0 versus 66.0±5.0 μM), but has a faster maximal turnover rate for the symmetric peptide (*k*_cat_ 8.7 × 10^−3^±0.3 × 10^−3^ versus 4.6 × 10^−3^±0.7 × 10^−3 ^s^−1^), resulting in similar *k*_cat_/*K*_M_ values. All *k*_cat_ values were lower and *K*_M_ were higher than those for the established H3K9me3 peptide substrate for KDM4E. The monomethylated H3K9Rme peptide had a higher *K*_M_ value than both the dimethylated peptides, and displayed the lowest turnover rate (*k*_cat_ 2.7 × 10^−3^±0.2 × 10^−3 ^s^−1^. Note that monomethylated lysine residues are poor substrates for the KDM4 subfamily^20^). For KDM5C, although both the asymmetric and symmetric dimethylarginine-containing peptides had similar *K*_M_ values (*K*_M_ 29.0±0.5 versus 21.1±5.8 μM), the turnover rate for the asymmetric peptide was an order of magnitude higher (41.2 × 10^−3^±3.5 × 10^−3^ versus 4.6 × 10^−3^±0.7 × 10^−3 ^s^−1^).

For KDM3A, the monomethylated arginine peptide was a better substrate than the corresponding dimethylated arginine peptides (Table 1). These results reflect the MS screening results, where in the context of overall incomplete substrate peptide demethylation, only conversion to the unmethylated residue was observed, that is, no monomethylated peptide intermediate was detected (Supplementary Fig. 3). Although the monomethylated arginine-methylated substrate showed a lower *k*_cat_ value than the lysine-methylated substrate (11.0±1.2 versus 355±95.3 s^−1^), the *K*_M_ values for all methylated arginine peptides were found to be smaller than those for H3K9me2 (H3K9Rme=12.1±3.1 μM, H3K9Rme2a=11.6±1.4 μM, H3K9Rme2s=23.7±1.9 μM and H3K9me2=44.5±4.8 μM). The *k*_cat_ value for K9Rme2a and K9Rme2s were lower than that for K9Rme. The combined kinetic results described above for each enzyme imply that the relative efficiency of RDM versus KDM substrate binding and rate of catalysis is sequence and context-dependent.

### RDM activity is observed on ‘natural' histone peptides

**Figure 4:**
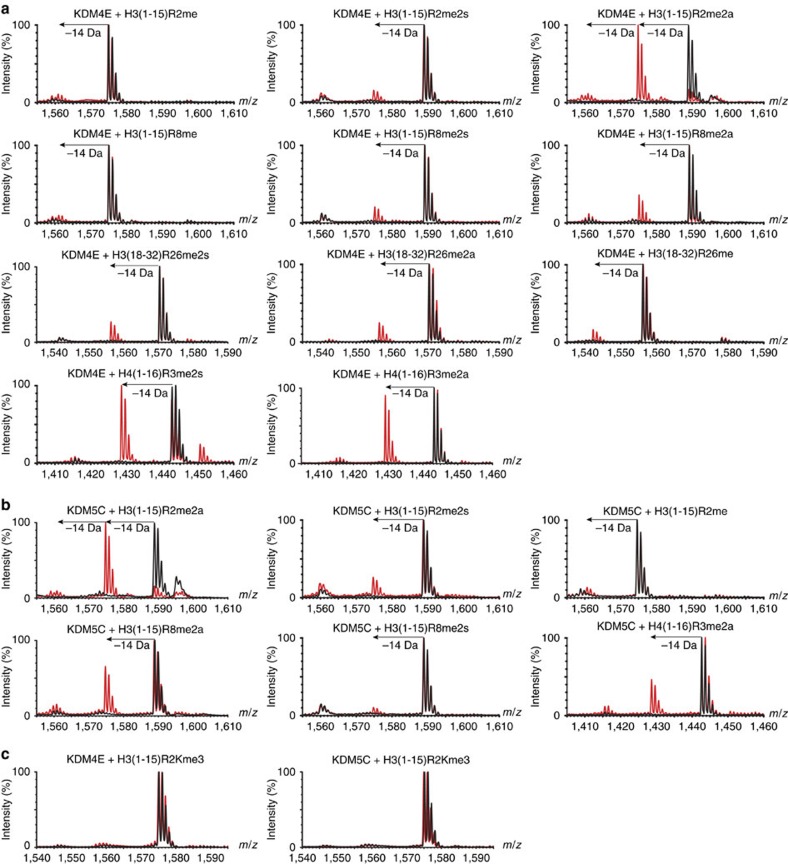
KDMs catalyse arginine demethylation in ‘natural' histone peptides. MALDI-TOF MS of demethylation of arginine methylated ‘natural' histone peptides by (**a**) KDM4E and (**b**) KDM5C. (**c**) MALDI-TOF MS revealing no demethylation activity of KMD4E or KDM5C on a H3(1–15)R2Kme3 peptide.

Given the ability of JmjC enzymes to catalyse demethylation of arginine residues in synthetic peptide sequences, we were interested to investigate whether these enzymes could also catalyse arginine demethylation in ‘natural' positions in histone peptides. Peptides representing known methylated arginine sites within the canonical histone tail sequences were synthesized and screened as substrates for the four enzymes identified as RDMs (Supplementary Table 1). Interestingly, arginine demethylation activity on the ‘natural' histone peptide sequences was observed for some, but not all of the KDMs (Table 2 and Fig. 4). Both KDM4E and KDM5C catalysed demethylation of some of the screened peptides, including peptides methylated at H3R2 and H4R3 (Fig. 4a,b and Supplementary Figs 8 and 9), while KDM3A and KDM6B displayed no activity on any of the new peptides tested (Supplementary Figs 10 and 11). The observation of a lack of RDM activity for KDM6B with these substrates is notable, given that KDM6B displayed such high RDM activity with the unnatural H3K27Rme2a substrate peptide (Table 1). Thus, these results demonstrate that RDM activity is dependent on the sequence context.

Further kinetic characterization was carried out on some of the ‘biologically relevant' peptides identified to be substrates for the KDMs (Table 1). Interestingly, despite the different neighbouring peptide sequences to the characterized lysine H3K9me3 substrate of KDM4E, the H3R2me2a peptide was found to be a better substrate for KDM4E than H3K9Rme2a or H3K9Rme2s (*k*_cat_/*K*_M_ 114.0 × 10^−6^ versus 70.4 × 10^−6 ^μM^−1 ^s^−1^/85.9 × 10^−6 ^μM^−1 ^s^−1^). The H4R3me2a peptide was a less efficient substrate for KDM4E, with a turnover rate almost five times lower than that of the H3R2me2a peptide, similar to that observed for H3K9Rme. For KDM5C, the H3R2me2a peptide had a lower *K*_M_ value than the H3K4Rme2a peptide, but was an overall slightly less efficient substrate for the enzyme (*k*_cat_/*K*_M_ 1,420 × 10^−6^ versus 630 × 10^−6 ^μM^−1 ^s^−1^). Nevertheless, with a *k*_cat_/*K*_M_ value only 14 times lower than that for H3K4me3 it appears possible that this reaction could also occur in cells in some contexts, particularly if promoted by auxiliary binding domains or binding partners (as is known to be important for some JmjC KDM activities (for example, PHF8))^24^.

Because some JmjC KDMs were found to catalyse demethylation of methylated arginine peptides in sequences substantially different from their characterized lysine substrates (for example, we observed low levels of demethylation of H4R3me2a (SG-Rme2a-GKGGKGLGKGGAK) by both KDM4E and KDM5C, Fig. 4), we were interested to see whether substitution of an arginine residue in the ‘natural' histone sequence with a methylated lysine residue would produce a KDM substrate. Interestingly, despite a peptide containing H3R2me2a being a relatively good RDM substrate for both KDM4E and KDM5C, the corresponding H3R2Kme3 peptide (A-Kme3-TKQTARKSTGGKA) was not observed to be a KDM substrate for either enzyme within our limits of detection (Fig. 4c). Overall, the combined results reveal that, at least for some JmjC demethylases, the preferred sequence contexts can differ for RDM and KDM activities.

**Figure 5:**
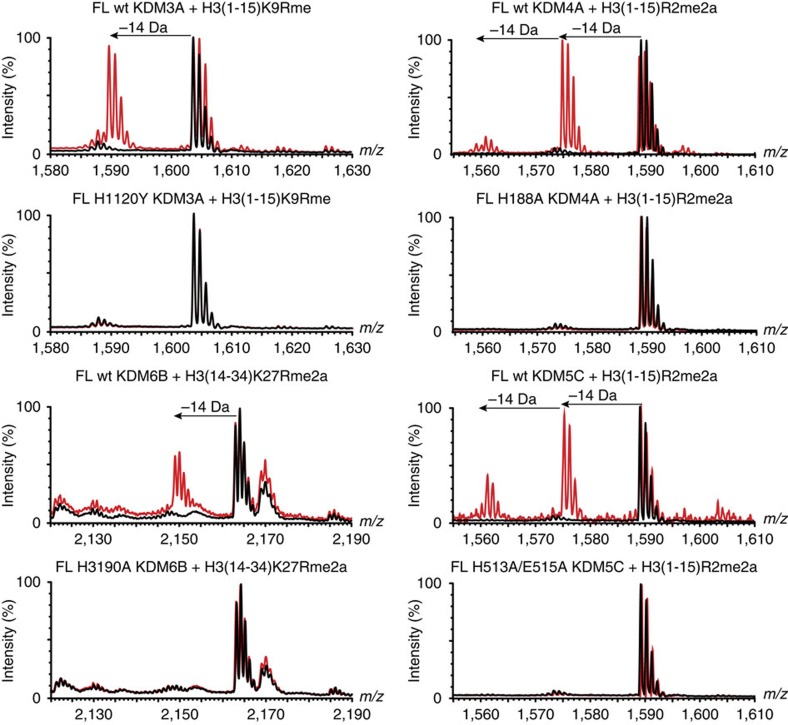
Full-length (FL) KDMs catalyse demethylation of arginine residues. MALDI-TOF MS of demethylation of arginine methylated peptides by Flag-tagged FL KDMs immunoprecipitated from HEK293T cells.

To investigate whether methylarginine demethylation can be catalysed by full-length JmjC KDMs, as well as by the truncated constructs, full-length proteins were exogenously overexpressed in HEK293T cells either as *N*-terminally Flag-tagged wild-type proteins or active-site variants known to be catalytically inactive^25^,^26^,^27^. The resultant full-length proteins were immunoprecipitated by their Flag-tags and used in MS assays with methylated histone peptides. As with the catalytic domain constructs, arginine demethylation was also observed with all four of the full-length proteins (KDM3A, KDM4A, KDM5C and KDM6B; note KDM4A was used in place of KDM4E as KDM4E is a pseudogene) tested on both ‘unnatural' and ‘natural' peptide substrates (Fig. 5 and Supplementary Figs 12–16). No RDM activity was observed with the catalytically inactive enzyme variants. Note that, with KDM3A, arginine demethylation activity was observed for the monomethylated peptides, but not for the symmetrically or asymmetrically methylated peptides; this result is consistent with the observed relative reaction efficiencies of these peptides with the truncated construct (Supplementary Fig 13 and Table 1).

### JmjC enzymes bind arginine and lysine in a similar manner

Crystallographic studies were then undertaken to investigate the binding modes of methylarginine-containing peptides with JmjC enzymes. KDM4A, a close structural homologue of KDM4E that can also catalyse demethylation of methylarginine peptides (albeit less efficiently in our current assays, Fig. 5), was selected for the crystallographic work because of its relatively robust crystallization properties and because it has previously been crystallized with *N*^ɛ^-methylated lysine peptide substrates^20^,^28^,^29^,^30^. A suitable crystal was obtained using KDM4A and an H4R3me2s fragment sequence (residues 1–15) with nickel substituting for iron and *N*-oxalylglycine (NOG) acting as a 2OG mimetic). The structure was solved by molecular replacement using PDB ID: 2OX0 as the search model; two molecules of KDM4A (chains A and B) are present in the asymmetric unit. In the initial electron density maps, other than for the methylated arginine itself, only weak difference in electron densities were observed for residues in the substrate peptide, suggesting that the mode of methylated arginine peptide binding could be flexible. We generated an *F*_*o*_–*F*_*c*_ OMIT map (where different densities were calculated omitting H4R3me2s) as a guide to locate any unmodelled difference peak around the KDM4A (A/B) active sites. This unweighted omit map revealed more than one likely conformation of the methylated arginine side chains and traces of H4R3me2s peptide backbones; only major conformations were modelled and refined in the final structure.

**Figure 6:**
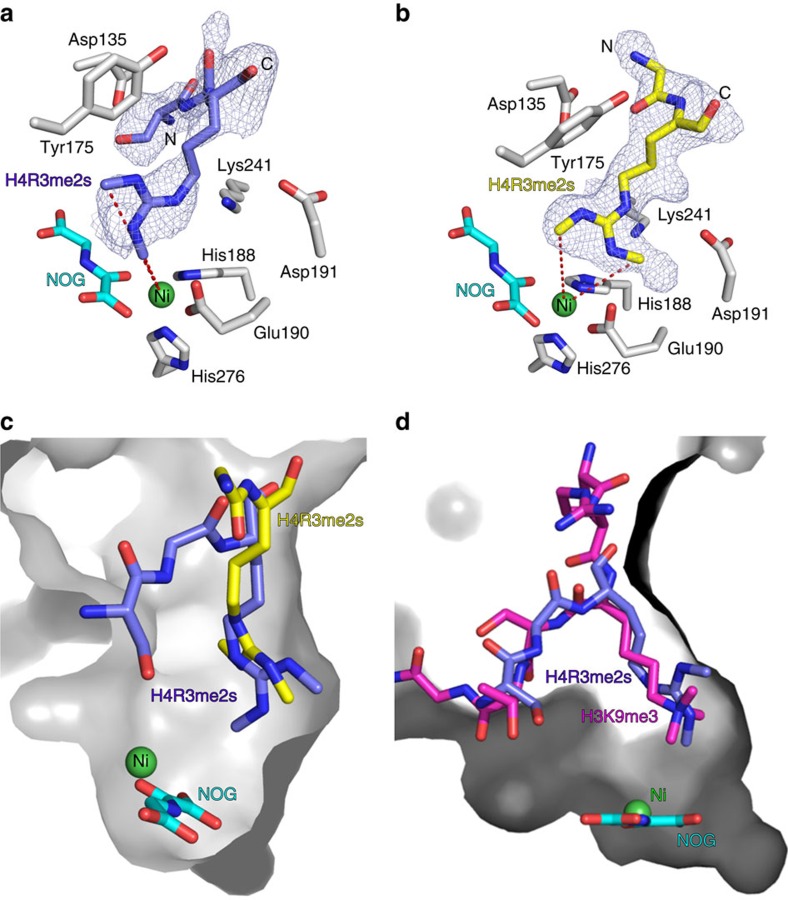
JmjC KDMs bind methylated arginine peptides in a catalytically-productive binding mode. (**a** and **b**) Views from an X-ray crystal structure of KDM4A in complex with nickel, NOG (a 2OG mimetic) and an H4R3me2s peptide (residues 1–15). Two orientations of peptide binding were refined; one orientation (**a**) positions a methyl group of symmetric dimethylarginine residue sufficiently close to the metal centre to allow catalysis (within 4.5 Å, for crystallographic reasons, nickel was substituted for iron). The other orientation (**b**) is likely not catalytically productive. Fo-Fc OMIT maps contoured to 3σ around the H4R3me2s residues are shown. (**c**) Overlay of the two binding orientations observed for the H4R3me2s peptide in the KDM4A active site. Only one orientation (blue) positions a methyl group close to the catalytic metal centre. (**d**) Overlay of the H4R3me2s peptide (catalytically-productive orientation only) and an H3K9me3 peptide (residues 7–14) bound in the KDM4A active site. The methylated arginine and lysine residues show similar binding modes.

Although care should be taken in over-interpreting ‘static' crystal structures of substrate fragments, it is of interest that the arginine side chain was observed to adopt two different extended conformations after refinement. One orientation positions one of the methyl groups of the symmetrically dimethylated arginine residue sufficiently close to the metal centre to enable catalysis (within 4.5 Å; Fig. 6a). The other side chain orientation is a likely catalytically unproductive conformation, wherein the side chain is oriented away from the metal (Fig. 6b; for overlay, see Fig. 6c). The presence of such an unproductive binding conformation might in part account for the higher degree of uncoupled 2OG turnover stimulated by methylated arginine compared with methylated lysine peptide binding, as observed for KDM6B with H3(14–34)K27Rme2a (for overlay of arginine and lysine binding, see Fig. 6d).

### Potential for JmjC RDM activity on non-histone substrates

**Figure 7:**
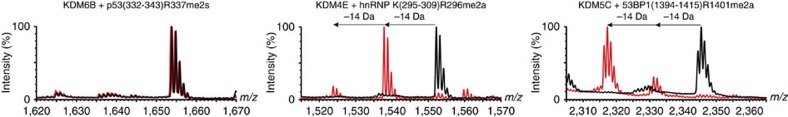
KDMs catalyse demethylation of arginine residues in non-histone substrates. MALDI-TOF MS assays for demethylation of non-histone arginine methylated peptides by KDMs. Red spectra show reactions including enzyme and black spectra the no-enzyme controls.

Arginine methylation has been reported for numerous non-histone proteins, as observed by antibody- and/or MS-based approaches, where it is proposed to regulate many cellular processes including alteration of cellular localization, protein stability and protein–protein/DNA interactions^31^,^32^. Although limited at present, there are some reports providing at least preliminary evidence for non-histone protein lysine demethylation catalysed by JmjC KDMs^33^,^34^,^35^. Given the ability of the JmjC KDMs to act as RDMs for peptides with sequences substantially different from their characterized lysine sequences, we were interested to investigate whether removal of non-histone arginine methylation could also be catalysed by 2OG oxygenases. As KDM6B is known to interact with tumour protein p53 (p53), peptide sequences containing known arginine methylation sites were synthesized and tested as JmjC RDM substrates^36^; however, no demethylation was observed (Fig. 7 and Supplementary Fig. 17). A selection of peptides from some other nuclear proteins known to be abundantly methylated on arginine residues were also synthesized and tested as RDM substrates by MS. While the majority of these peptides were found not to be substrates, in some cases clear arginine demethylation was observed (Fig. 7 and Supplementary Fig. 17). Demethylation of asymmetric dimethylated arginine residues within peptides from heterogeneous nuclear ribonucleoprotein K (hnRNP K) and tumour suppressor p53-binding protein 1 (53BP1) was observed, as catalysed by KDM4E and KDM5C, respectively. If replicable in cells, these results raise the possibility that arginine demethylation could be a widespread phenomenon extending well beyond the canonical histone tails.

## Discussion

Since the observation of *N*^ɛ^-lysine methylation in 1959 (ref. 37), multiple protein residues have been found to undergo *N*- and *O*-methylation^31^,^38^. Within the context of gene regulation, the *N*-methylation of lysine and arginine residues on histone tails plays critical roles. Multiple methyltransferases have been identified, many of which are apparently highly promiscuous, acting to catalyse methylation on numerous proteins^31^,^33^,^39^,^40^. Despite the pioneering observations in 1973 of the oxidative demethylation of *N*^ɛ^-methylated lysine residues in calf thymus histones^41^, the identification of the sequences of *N*^ɛ^-methyl-group demethylases took three decades. Following the identification of KDM1A in 2004 (ref. 42), the subsequent discovery of the JmjC KDMs validated the early studies in identifying oxygenases that catalyse *N*^ɛ^-methyllysine demethylation to release formaldehyde^4^,^43^,^44^. Given the plethora of methylated proteins, it appears possible that some KDMs might be found to be equally promiscuous as KMTs.

The possibility of RDM activities related to those of the KDMs is readily envisaged. While no lysine-specific demethylase-type flavin-dependent RDMs have been reported (although are feasible), the JmjC oxygenase JMJD6 has been reported as an RDM acting on histones and other non-histone proteins^9^,^15^,^16^. However, in our laboratory's work with JMJD6 we have observed only lysine hydroxylation, not RDM activity^10^,^45^. Various reports have appeared in the recent literature describing both hydroxylation and RDM activities for JMJD6 (refs 13, 14, 15, 16, 17, 46). Other JmjC oxygenases have also been the subject of controversial assignments^47^,^48^,^49^. In most cases the cell-based work has principally employed antibody-based methods, in some cases supported by ‘proteomic' MS evidence, and at present biochemical evidence for the RDM activity of JMJD6 remains limited. Identification of both RDM and KDM activities in a cellular context using antibodies can be complicated by difficulties in accurately quantifying absolute levels of both methylated substrates and the demethylated products and (along with other factors) by a lack of appropriately specific antibodies for substrates and products^50^. MS-based methods, while rapidly improving, are also not normally sufficiently well optimized for measuring absolute levels in cells, particularly for low abundance post-translational modifications^32^,^50^,^51^,^52^. Thus, while we appreciate that enzyme activity may differ in the isolated protein form from cellular contexts (for example, as observed for the 2OG-dependent protein hydroxylase factor inhibiting hypoxia-inducible factor^53^), given the complexity and controversy in JmjC assignments, in particular, in the arginine demethylation field, we believe that, where possible, it is important to define the ‘biochemical reactions' catalysed by potential RDMs (and KDMs) using isolated enzymes.

The assays applied here directly measure, either by mass shifts or by the appearance of new NMR signals, the product(s) of KDM/RDM activity. It should be noted that in the context of MS analyses we undertook to carry out careful non-enzyme controls to ensure that the conditions of MS analysis did not invoke apparent demethylation, or that we were not observing contamination of the putative product in the starting material, which might not be apparent before incubation.

The results clearly show that, at least under our assay conditions, some, but not all, recombinant forms of JmjC KDMs do have RDM activity. Thus, we observed that KDM3A, KDM4E, KDM5C and KDM6B can catalyse demethylation of methylated arginine residues when substituted for the methylated lysine residue in their characterized peptide substrates. In addition, KDM4E (and full-length KDM4A) and KDM5C were found to catalyse demethylation of histone peptides methylated at H3R2, H3R8, H3R26 (KDM4E, but not KDM5C) and H4R3, and also some non-histone peptide sequences.

One interesting result to emerge from the studies on both histone and non-histone RDM and KDM substrates for the JmjC enzymes is that the apparent sequence selectivities can differ for RDM and KDM activities, even with the same enzyme. Thus, for example, H3R2me2a is a better substrate for KDM4E than H3K9Rme2a (Table 1), and the analogous H3R2Kme3 peptide is not a substrate for KDM4E or KDM5C (Fig. 4c). Although the biological significance of these results is presently unclear, they can be rationalized by the likelihood that in order for productive demethylation to occur, the methylarginine and methyllysine side chains are likely accommodated differently by dual-function KDM/RDM enzymes in order to ensure a catalytically productive relationship between the reactive Fe(IV)=O intermediate and the methyl group at the active site. The differential orientation of the lysine and arginine side chains may in turn have ‘knock-on' effects with respect to optimal binding of the surrounding substrate residues. Support for this proposal comes from work revealing that in the case of KDM4A-C, aside from the methylated lysine residues, the H3K9 and H3K36 substrates are accommodated differently at the entrance to the active site^30^,^54^. Given that for some KDMs post-translational modifications distinct from the residue undergoing demethylation can substantially promote activity, it is possible that RDM activity is similarly promoted. The partial decoupling of 2OG oxidation and demethylation observed for some RDM substrates may reflect non-optimal substrate sequences, or use of a truncated substrate, as precedented with other 2OG-dependent protein hydroxylases^55^. However, it is important to note that, at least in the case of KDM6B, some of the RDM activities we observe are of similar levels to the KDM activity, implying the likelihood of biological relevance of the dual-function KDM/RDM results in some situations (including by competition between substrates).

We appreciate that further work is required on protein substrates in cells for the full biological relevance of these results to be established. Despite considerable efforts, we are not yet able to conclusively determine RDM activity in cells, in part because of the lack of suitably useful antibodies. Nonetheless, the biochemical assignment of RDM activity is interesting and relevant to future work on the functional studies on JmjC enzymes in cells. The JmjC KDMs have been linked to a plethora of human developmental diseases and cancers, and there is growing interest in them as pharmaceutical targets^56^,^57^,^58^. In addition to helping to elucidate the regulatory mechanisms involved in arginine methylation cycles, these results reveal new enzymatic activity and function of the JmjC KDMs, an understanding of which may be crucial to their effective therapeutic targeting. In this regard, it is important to note that, at least some of the roles of JmjC KDMs in disease may involve their production of formaldehyde, which is a product of both KDM and RDM catalysis, and which, at least above threshold levels, is toxic^59^. Now that enzymes that have RDM activity have been conclusively identified, efforts can focus on the demonstration of their biological relevance.

## Methods

### Materials

Full-length Flag-tagged KDMs were expressed in HEK293T cells (ATCC) using plasmids encoding full-length KDM4A_1–1,064_ (ref. 26), H188A KDM4A_1–1,064_ (ref. 26), KDM6B_1–1,636_ (ref. 27), H1390A KDM6B_1–1,636_ (ref. 27), KDM3A_1–1,321_ (ref. 25) and H1120Y KDM3A_1–1,321_ (ref. 25) as described previously. Plasmids encoding Flag-tagged KDM5C_1–4,680_ and H513A/E515A KDM5C_1–4,680_ were kindly provided by Pavel Savitsky (Structural Genomics Consortium, Oxford). Recombinant constructs of KDM2A_1–557_ (ref. 60), KDM4A_1–359_ (ref. 30), KDM4E_1–337_ (ref. 61), KDM6B_1,141–1,590_ (ref. 62) and PHF8_1–447_ (ref. 63) were produced as *N*-terminally His_6_-tagged proteins in *Escherichia coli* cells. KDM3A_515–1,317_ was produced as an *N*-terminally His_6_-tagged protein in Sf9 cells^62^. KDM5C_1–765_ was produced as an *N*-terminally His_6_-tagged protein in Sf9 cells and was kindly provided by Aleksandra Szykowska (Structural Genomics Consortium). Proteins were purified by Ni-affinity chromatography and size exclusion chromatography as previously described.

### Peptide synthesis

Peptides were produced in house using a Liberty Blue automated microwave peptide synthesizer (CEM Corporation) for kinetic and crystallographic studies or either a Multipep RSi (Intavis Bioanalytical Instruments AG) or an S336X peptide synthesizer (CS Bio) for screening purposes. Fluorenylmethyloxycarbonyl-mediated solid-phase chemistry (on MBHA resin) was used to produce the peptides with *C*-terminal amides. After synthesis, peptides were cleaved from the resin by incubation with 92.5% trifluoroacetic acid/2.5% water/2.5% triisopropylsilane/2.5% dimethoxybenzene (3 h) followed by precipitation with ice-cold diethyl ether. Lyophilized peptides produced on the Liberty Blue or S336X synthesizers were purified by reverse-phase high-performance liquid chromatography using a Vydac C18 column (Solvent A=0.1% trifluoroacetic acid in H_2_O, Solvent B=0.1% trifluoroacetic acid in acetonitrile) to >95% purity as determined by MS. Peptides used for screening were assayed unpurified. Peptide sequences are given in Supplementary Table 1. Methylated arginines suitably protected for solid-phase peptide synthesis were purchased from Novabiochem. In all cases the observed masses for peptides were within 2 Da of the predicted values by MALDI MS.

### Activity assays

*MALDI MS activity assays*. Recombinant proteins (2 μM) were incubated with peptides (10 μM, sequences are given in Supplementary Table 1) and cofactors/substrates for 1 h at 37 °C, unless otherwise specified. Reactions were quenched with 1:1 (v/v) methanol and analysed with MALDI MS. Assay conditions for each enzyme are given in Supplementary Table 2. Negative controls without enzyme were included. Note that in some cases small variations (≤2 Da) in the absolute experimentally observed values compared with the calculated values for peptides were observed. However, in all cases where demethylation is assigned the mass shift was 14 Da.

*Formaldehyde dehydrogenase-coupled assays*. Preliminary kinetic parameters were determined by detection of the formaldehyde by-product of the demethylation reaction using a FDH (formaldehyde dehydrogenase-coupled)/NAD^+^-coupled assay as previously described^61^. Assays were carried out in buffer (enzyme-dependent) with the addition of Fe(II) ammonium sulfate (10 μM), sodium ascorbate (100 μM), 2OG (200 μM), NAD^+^ (500 μM), enzyme (0.2–4 μM) and FDH enzyme (0.025 U per assay, Sigma-Aldrich; total volume 30 μl) and were conducted at 37 °C in clear-bottom black 384-well plates. Reactions were monitored over 25 min using a PHERAstar FS (BMG Labtech) plate reader with 355 nm excitation and 460 nm emission. The *K*_m_ and *V*_max_ values were calculated from the reaction rate during the linear phase of formaldehyde production, as determined by the slope of the fluorescence signal recorded.

*NMR assays*. NMR spectroscopy was carried out as described^22^. NMR spectra were recorded using a Bruker Avance AVIII 700 MHz spectrometer equipped with an inverse TCI cryoprobe, optimized for ^1^H observation and installed with the Topspin software. All samples were prepared in Eppendorf tubes (75 μl volume) before being transferred to 2-mm MATCH NMR tubes (Wilgenberg); time course data were then collected over a period of 20 min at 89-s intervals using an automated routine. The solvent deuterium signal was used as an internal lock signal, and the solvent signal was reduced by excitation sculpting^64^. Enzyme stocks were in protiated HEPES/NaCl buffer, pH 7.5, which was diluted with dAFN buffer (prepared by diluting 500 mM ammonium formate, 5 M NaCl in H_2_0 pH 7.5 to 10% v/v in D_2_O) when added to the samples. Chemical shifts are reported relative to the solvent water resonance (δ_H_ 4.7 p.p.m.).

### Cell culture

HEK293T cells (ATCC) were cultured at 37 °C and 5% CO_2_ in high-glucose DMEM (Sigma) supplemented with 10% fetal bovine serum (Sigma) and 1% Glutamax (Sigma). Full-length KDMs with Flag-tags were transiently transfected into HEK293T cells using linear polyethylenimine (PEI) (10 μg DNA, 60 μl PEI per T75, 60% confluency, PAA Laboratories) and were harvested after 24–48 h.

### On-bead demethylation assays

Cells transiently transfected with the relevant KDM were washed in PBS buffer and lysed in 50 mM HEPES, pH 7.5, 150 mM NaCl, 0.5% NP40, 1% EDTA-free protease inhibitor cocktail and DNAse (10 μg ml^−1^). Lysate was diluted fivefold with buffer containing 50 mM HEPES, pH 7.5, 150 mM NaCl before being incubated with 20 μl anti-FLAG M2 Magnetic Beads (Sigma-Aldrich) for 3 h at 4 °C. Beads were washed four times with lysis buffer (500 μl) and two times with 50 mM HEPES (500 μl; or 50 mM HEPES, 150 mM NaCl where NaCl was used in the reaction buffer) before MS analysis. Beads were resuspended in a final volume of 30 μl for use in activity assays.

Demethylation reactions were conducted with 2 μl of the immobilized anti-FLAG bead suspension with peptide (10 μM) and cofactors/substrates (conditions given in Supplementary Table 3). Reactions were incubated for 3 h at 37 °C and quenched with 1:1 MeOH by volume. The demethylation products were detected by MALDI MS. Western blotting of lysate samples was carried out to confirm exogenous overexpression of all enzymes using mouse anti-Flag primary (F3165 Sigma, 1:1,000, overnight, 4 °C) and goat anti-mouse-HRP (W4021 Promega, 1:10,000, 1 h, room temperature, Supplementary Fig. 12).

### Crystallization

Co-crystals of KDM4A_1–359_.Ni(II).H4(1–15)R3me2s.NOG were obtained in sitting drops grown at 4 °C. Drops contained 10 μg ml^−1^ KDM4A, 5 mM H4(1–15)R3me2s, 5 mM NOG and 4 mM NiCl_2_. Crystals were cryoprotected with 25% glycerol before being flash-frozen in liquid N_2_. Data were collected using a single crystal at 100 K at the Diamond I04-1 MX beam line and were processed with HKL2000 (ref. 65). The structure was solved by molecular replacement using PHASER^66^ (search model PDB ID 2OX0) and was refined by alternative cycles of CNS^67^ and PHENIX^68^, with iterative rebuilding of the refined model using COOT^69^. All residues were in the allowed regions of the Ramachandran plot as calculated by PROCHECK^70^. Data collection and refinement statistics are given in Supplementary Table 4. A stereo image of a portion of the electron density map is shown in Supplementary Fig. 18.

### Statistical analysis

Each result shows the mean of three independent experiments with error bars representing the s.e.m. Each experiment was carried out (at least) in technical triplicate.

### Data availability

The data that support the findings of this study are available from the corresponding author upon request. The crystal structure has been deposited under PDB accession code 5FWE.

## Additional information

**How to cite this article:** Walport, L. J. *et al*. Arginine demethylation is catalysed by a subset of JmjC histone lysine demethylases. *Nat. Commun.* 7:11974 doi: 10.1038/ncomms11974 (2016).

## Supplementary Material

Supplementary Information Supplementary Figures 1-18, Supplementary Table 1-4 and Supplementary References

## Figures and Tables

**Figure 1:**
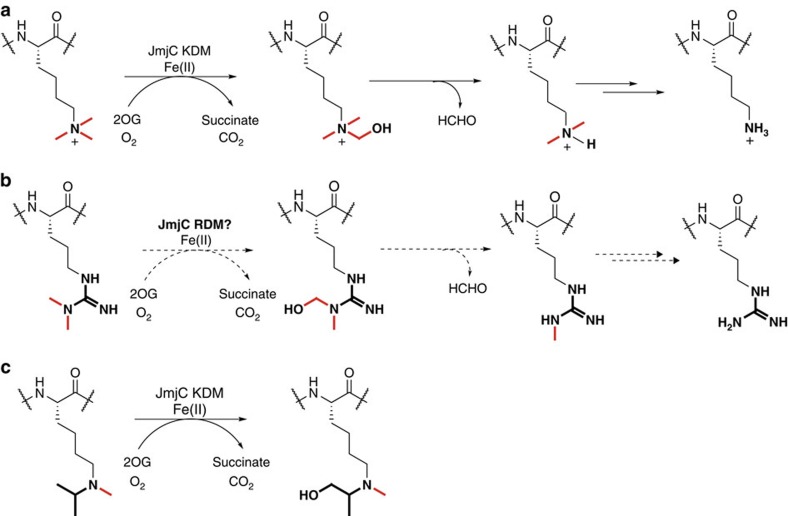
Mechanism of lysine and arginine demethylation. (**a**) JmjC lysine demethylases (KDM2–7 subfamilies) catalyse oxidative decarboxylation of 2OG to form succinate, carbon dioxide and a reactive iron(IV)-oxo intermediate; this intermediate then facilitates hydroxylation of the lysine *N*^*ɛ*^-methyl group to form an unstable hemiaminal. Fragmentation of the hemiaminal releases formaldehyde and the unmethylated lysine residue. (**b**) Proposed mechanism for JmjC-catalysed arginine demethylation. (**c**) Hydroxylation of the *N-*methyl,*N*-isopropyllysine derivative is catalysed by various JmjC KDMs at a position analogous to that required for demethylation of asymmetrically dimethylated arginine residues. Methyl groups are bold red lines, dashed arrows represent proposed reactions and HCHO is formaldehyde. Each JmjC KDM/RDM/oxidation reaction is coupled to the conversion of 2OG and oxygen to carbon dioxide and succinate.

**Table 1:** Kinetic parameters for arginine demethylation by KDMs (RDMs).

Enzyme	Substrate	*K*_M_/μM	*k*_cat_ × 10^−3 ^s^−1^	*k*_cat_/*K*_M_ × 10^−6^
KDM3A	**H3(1-15)K9me2**	44.5±4.8*	355±95.3*	**7,978.0**
	H3(1-15)K9Rme	12.1±3.1	11.0±1.2	907.6
	H3(1-15)K9Rme2a	11.6±1.4	5.0±0.6	430.7
	H3(1-15)K9Rme2s	23.7±1.9	3.0±0.3	126.5
KDM4E	**H3(1-15)K9me3**	**17.8±4.0**	**170.7±15.2**	**9,575**
	H3(1-15)K9Rme2a	66.0±5.0	4.6±0.7	70.4
	H3(1-15)K9Rme2s	101.1±11.0	8.7±0.3	85.9
	H3(1-15)K9Rme	168.8±35.9	2.7±0.2	15.9
	*H3(1-15)R2me2a*	*86.9*±*16.5*	*9.9*±*1.0*	*114.0*
	*H4(1-15)R3me2a*	*121.5*±*10.1*	*2.1*±*0.1*	*17.7*
KDM5C	**H3(1-15)K4me3**	**7.6±2.9**	**67.1±10.3**	**8,800**
	H3(1-15)K4Rme2a	29.0±0.5	41.2±3.5	1,420.0
	H3(1-15)K4Rme2s	21.1±5.8	4.6±0.7	219.0
	*H3(1-15)R2me2a*	*19.0*±*1.9*	*12.0*±*0.7*	*630.0*
KDM6B	**H3(14-34)K27me3**	**6.7±0.6**	**11.7±0.1**	**1,750.0**
	H3(14-34)K27Rme2a	676.3±118.3	28.8±3.6	42.5

KDM, *N*^ɛ^-methyllysine demethylase; RDM, *N*^ω^-methylarginine demethylase.

Apparent kinetic parameters (*K*_M_ and *k*_cat_ values) were determined for each JmjC KDM/RDM using a formaldehyde dehydrogenase-coupled assay. Data show mean±s.e.m (*n*=3). Recognized histone *N*^ɛ^-methylated-lysine peptide substrate for each KDM is marked in bold. ‘Natural' histone peptides are shown in italics.

*k*_cat_/*K*_M_ values are calculated form the average *k*_cat_ and *K*_M_ values for each peptide.

* Kinetic parameters calculated using data points below 100 μM, substrate inhibition observed above 100 μM.

**Table 2:** Summary of observed arginine demethylation activity of tested RDMs.

KDM	H3 (1–15) R2X			H3 (1–15) R8X			H3 (10–24) R17X			H3 (18–32) R26X			H4 (1–15) R3X		
	me	me2a	me2s	me	me2a	me2s	me	me2a	me2s	me	me2a	me2s	me	me2a	me2s
KDM3A	−	−	−	−	−	−	−	−	−	−	−	−	−	−	−
KDM4E	+	+	+	+	+	+	−	−	−	+	+	+	−	+	+
KDM5C	+	+	+	−	+	+	−	−	−	−	−	−	−	+	−
KDM6B	−	−	−	−	−	−	−	−	−	−	−	−	−	−	−

+ indicates demethylation activity; − indicates no activity or that below our limits of detection; KDM, *N*^ɛ^-methyllysine demethylase; RDM, *N*^ω^-methylarginine demethylase. X corresponds to me/me2a/me2s.
