# Supplementary material for: Arginine demethylation is catalysed by a subset of JmjC histone lysine demethylases
Source: Nat Commun. 2016 Jun 23;7:11974. doi: 10.1038/ncomms11974 (PMC4931022; doi:10.1038/ncomms11974)
Supplement: Supplementary Information — Supplementary Figures 1-18, Supplementary Table 1-4 and Supplementary References [file ncomms11974-s1.pdf]

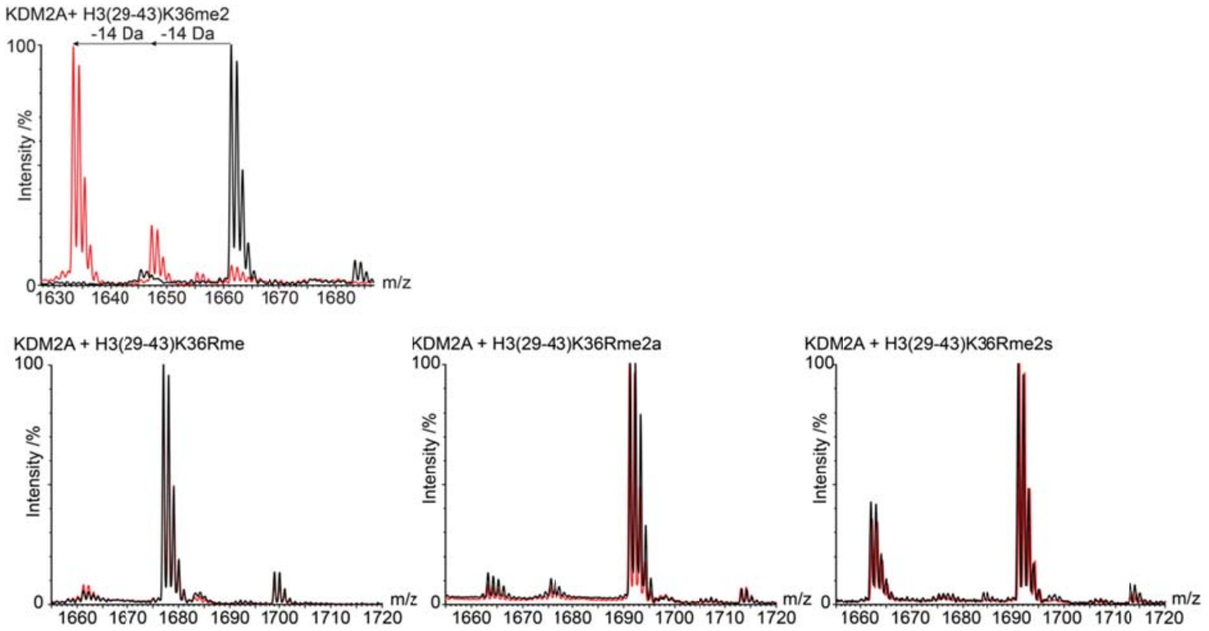

Supplementary Figure 1 KDM2A catalyses only lysine demethylation. MALDI-TOF MS of demethylation of the shown variant histone peptides as catalysed by recombinant KDM2A. Reactions containing enzyme are in red with peptide only controls in black.

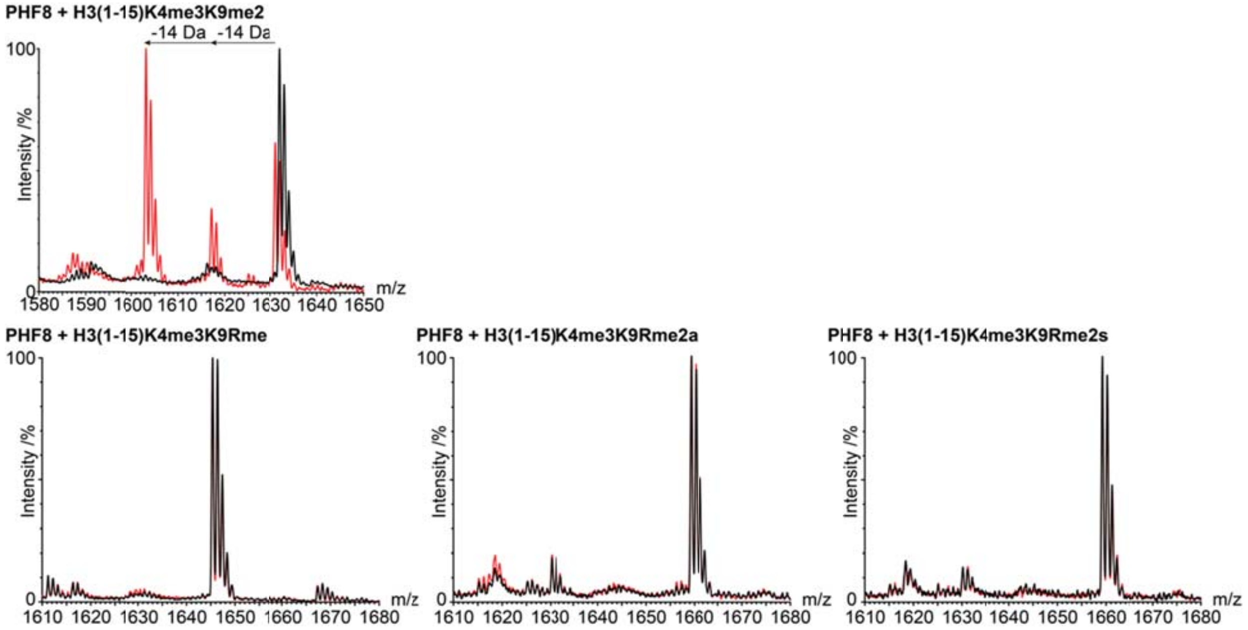

Supplementary Figure 2 PHF8 catalyses only lysine demethylation. MALDI-TOF MS of demethylation of the shown variant histone peptides as catalysed by recombinant PHF8. Reactions containing enzyme are in red with peptide only controls in black.

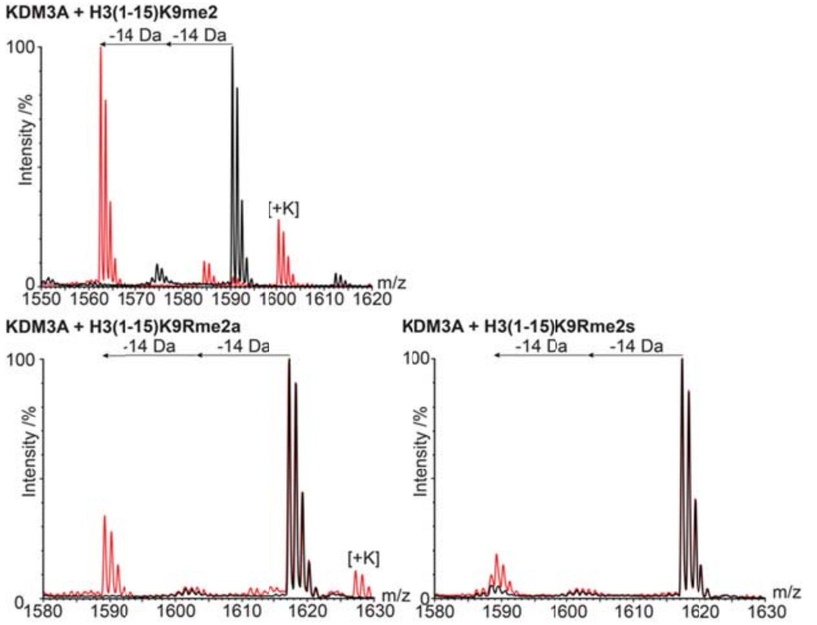

Supplementary Figure 3 KDM3A catalyses lysine and arginine demethylation. MALDI-TOF MS of demethylation of the shown variant histone peptides as catalysed by recombinant KDM3A. Reactions containing enzyme are in red with peptide only controls in black. See main text for other KDM3A assays (Fig. 2b).

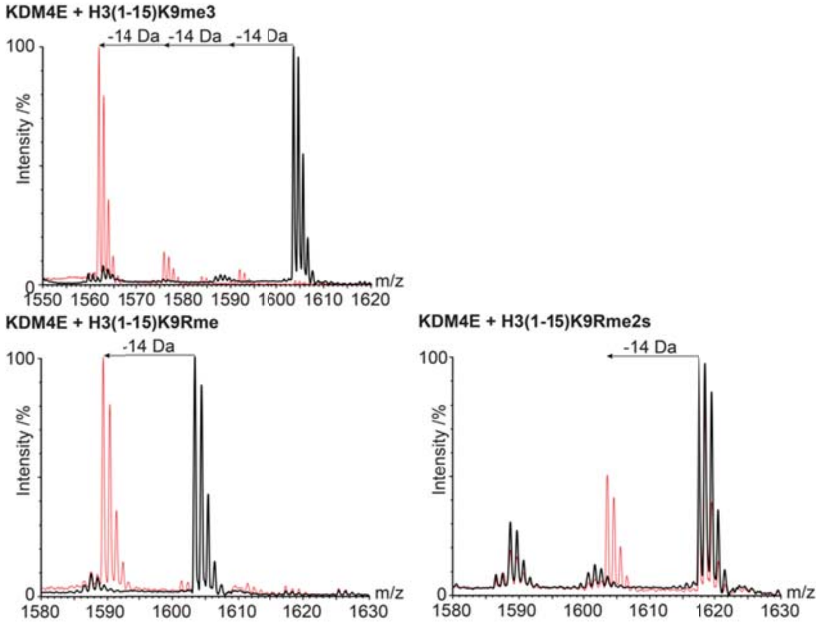

Supplementary Figure 4 KDM4E catalyses lysine and arginine demethylation. MALDI-TOF MS of demethylation of the shown variant histone peptides as catalysed by recombinant KDM4E. Reactions containing enzyme are in red with peptide only controls in black. See main text for other KDM4E assays (Fig. 2b).

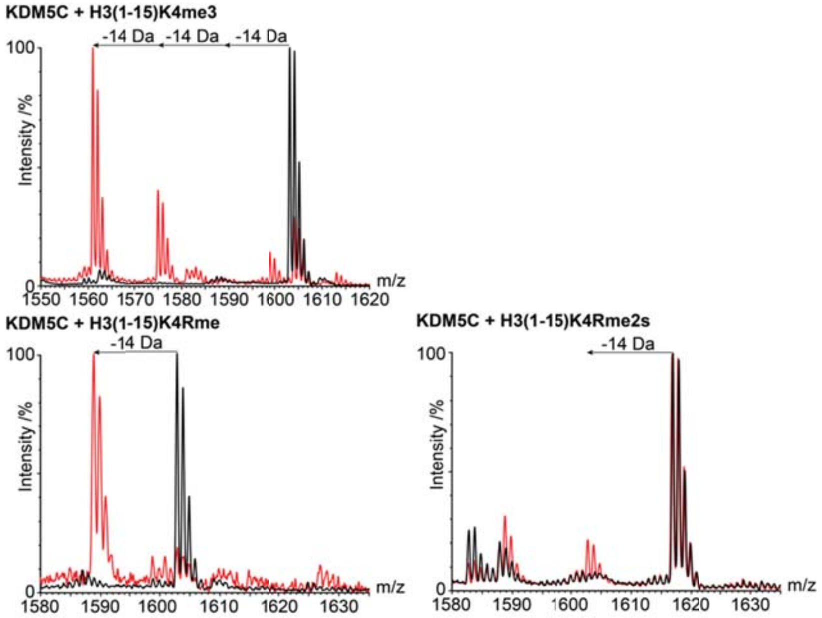

Supplementary Figure 5 KDM5C catalyses lysine and arginine demethylation. MALDI-TOF MS of demethylation of the shown variant histone peptides as catalysed by recombinant KDM5C. Reactions containing enzyme are in red with peptide only controls in black. See main text for other KDM5C assays (Fig. 2b).

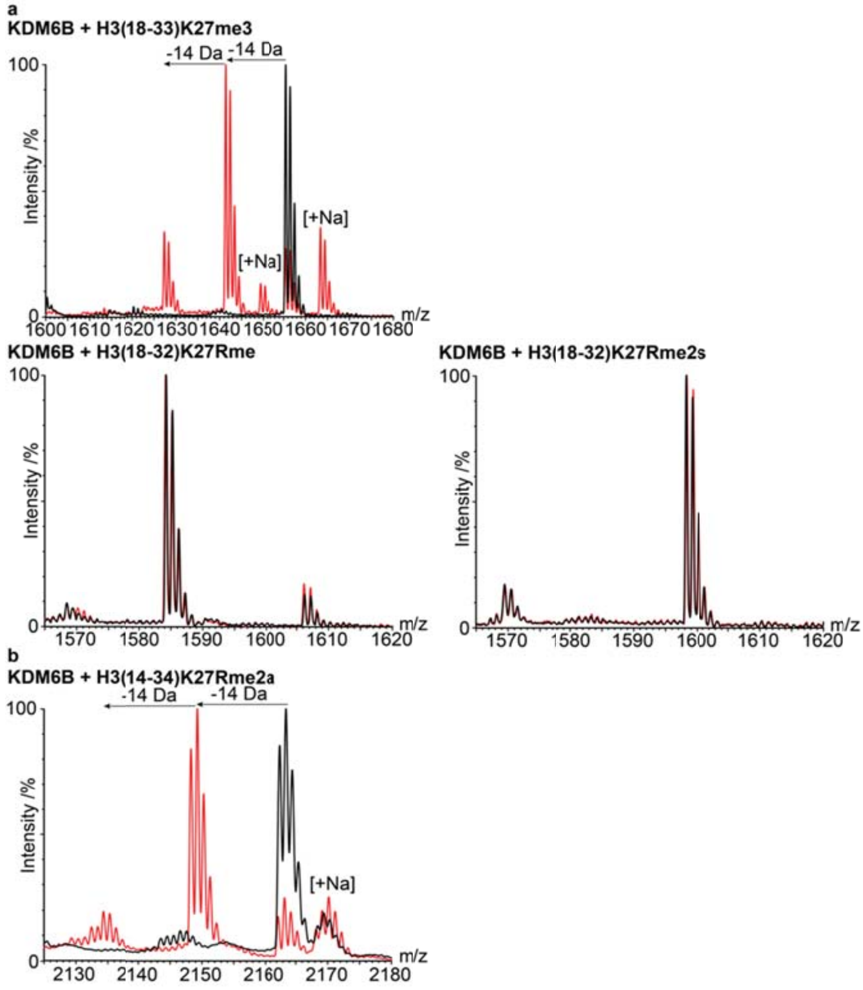

Supplementary Figure 6 KDM6B catalyses lysine and arginine demethylation. MALDI-TOF MS of demethylation of the shown variant histone peptides as catalysed by recombinant KDM6B. Reactions containing enzyme are in red with peptide only controls in black. (a)

reactions with 15mer arginine methylated-peptides (16mer lysine methylated-peptide) (b) reaction with 21mer peptide. See main text for other KDM6B assays (Fig. 2b).

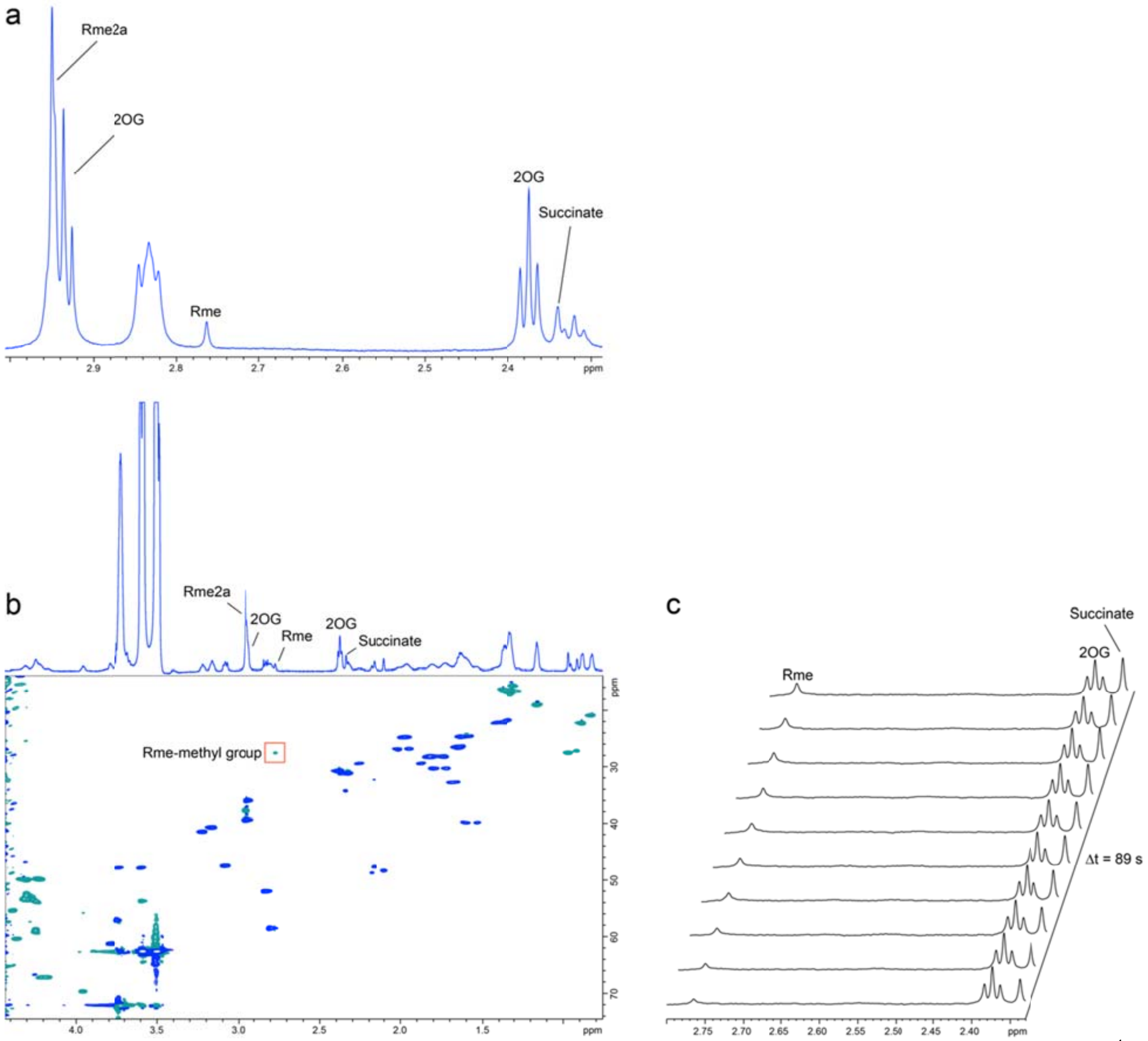

Supplementary Figure 7 NMR analysis of KDM6B catalysed arginine demethylation. (a) ^{1}H NMR spectrum of KDM6B-catalysed demethylation of a 15 residue H3K27me2a peptide (residues 18-32, sequence KQLATKAARRme2aSAPAT). ^{1}H-resonances for 2OG, succinate and the arginine methyl groups of substrate and monomethylated product peptide (δ_H 2.76 ppm) are highlighted. (b) ^{1}H-^{13}C-HSQC spectrum of KDM6B-catalysed demethylation of an H3K27Rme2a peptide (residues 18-32, sequence KQLATKAARRme2aSAPAT). The peak corresponding to the methyl group of N^{ω}-monomomethylarginine (δ_H 2.76 ppm, δ_C 27.5 ppm) is highlighted. The formaldehyde scavenger dimedone is present in the sample. (c) ^{1}H spectra showing time-dependent formation of N^{ω}-monomomethylarginine and succinate during the KDM6B-catalysed demethylation of a 21 residue H3K27Rme2a peptide (residues 14-34, sequence KAPRKQLATKAARRme2aSAPATGG).

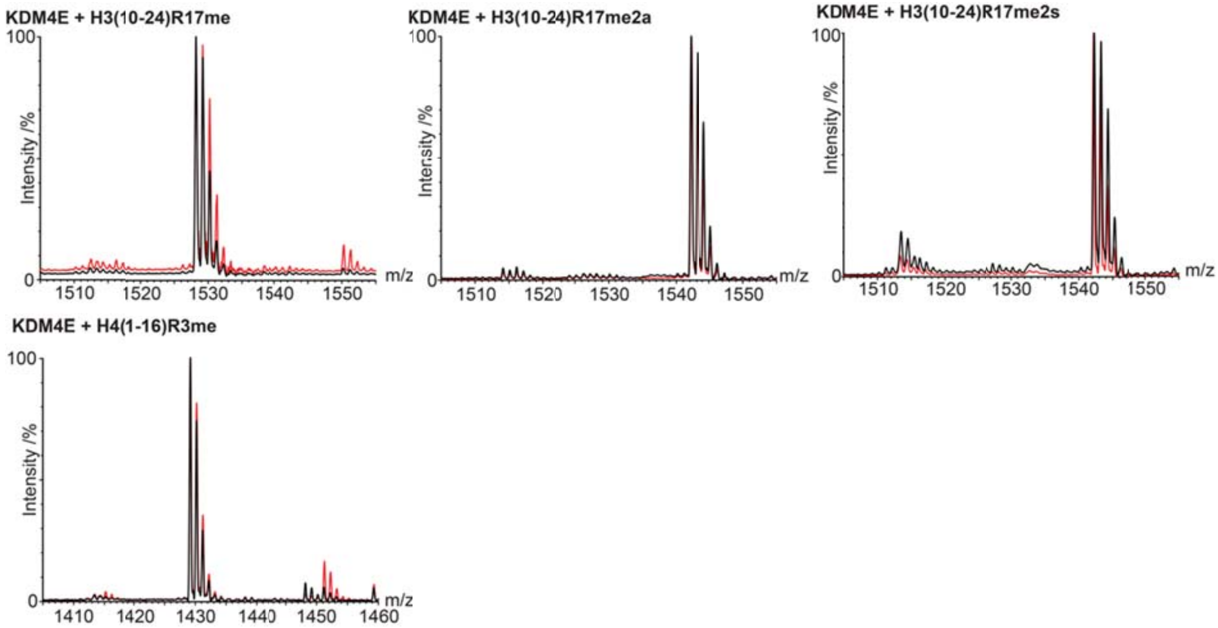

Supplementary Figure 8 KDM4E catalyses arginine demethylation in histone peptides. MALDI-TOF MS of demethylation of the shown arginine methylated histone peptides as catalysed by recombinant KDM4E. Reactions containing enzyme are in red with peptide only controls in black See main text for other assays (Fig. 4a).

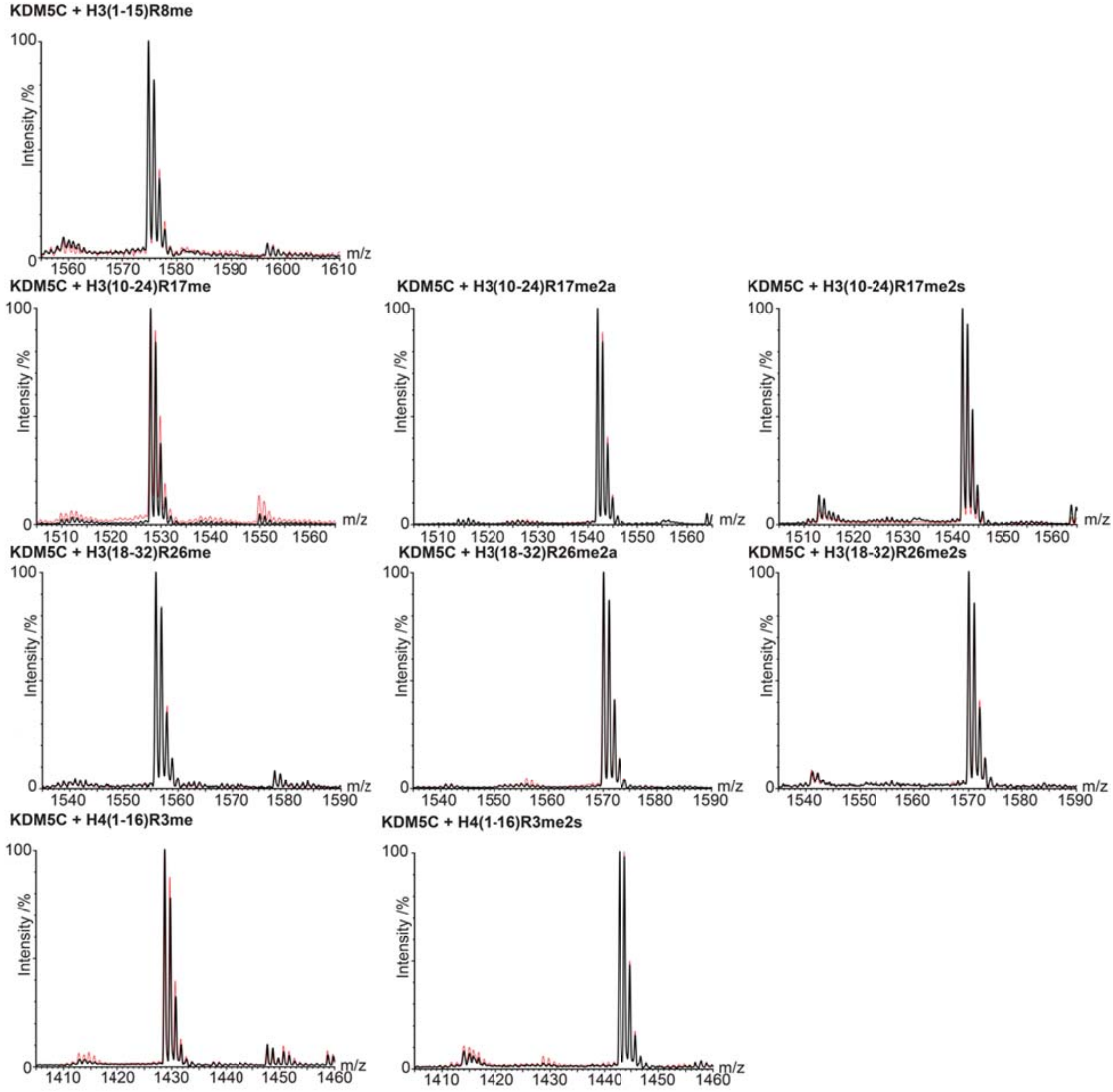

Supplementary Figure 9 KDM5C catalyses arginine demethylation in histone peptides. MALDI-TOF MS of demethylation of the shown arginine methylated histone peptides as catalysed by recombinant KDM5C. Reactions containing enzyme are in red with peptide only controls in black See main text for other assays (Fig. 4b).

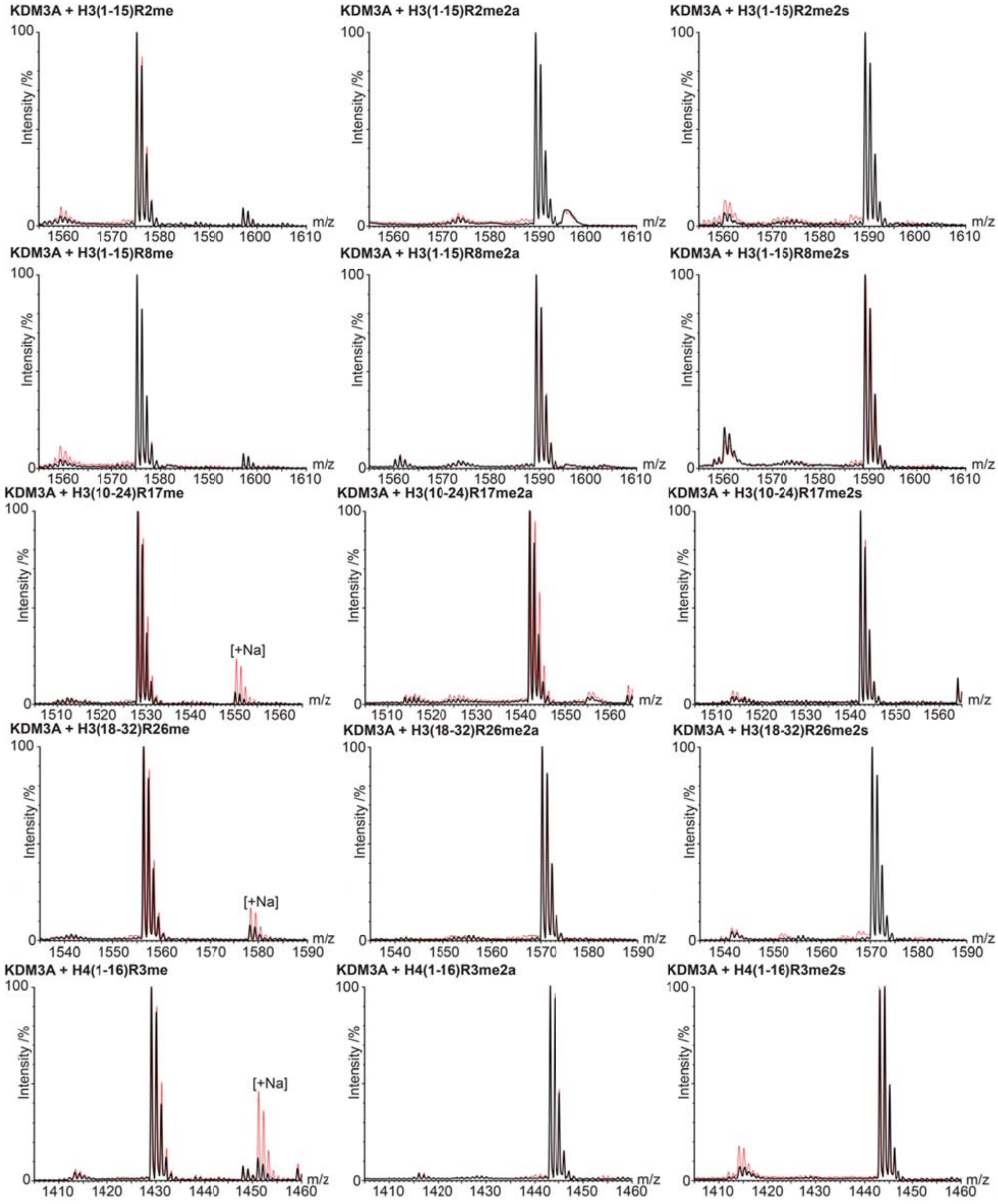

Supplementary Figure 10 KDM3A does not catalyse histone arginine demethylation. MALDI-TOF MS of demethylation of the shown arginine methylated histone peptides catalysed by recombinant KDM3A. Reactions containing enzyme are in red with peptide only controls in black.

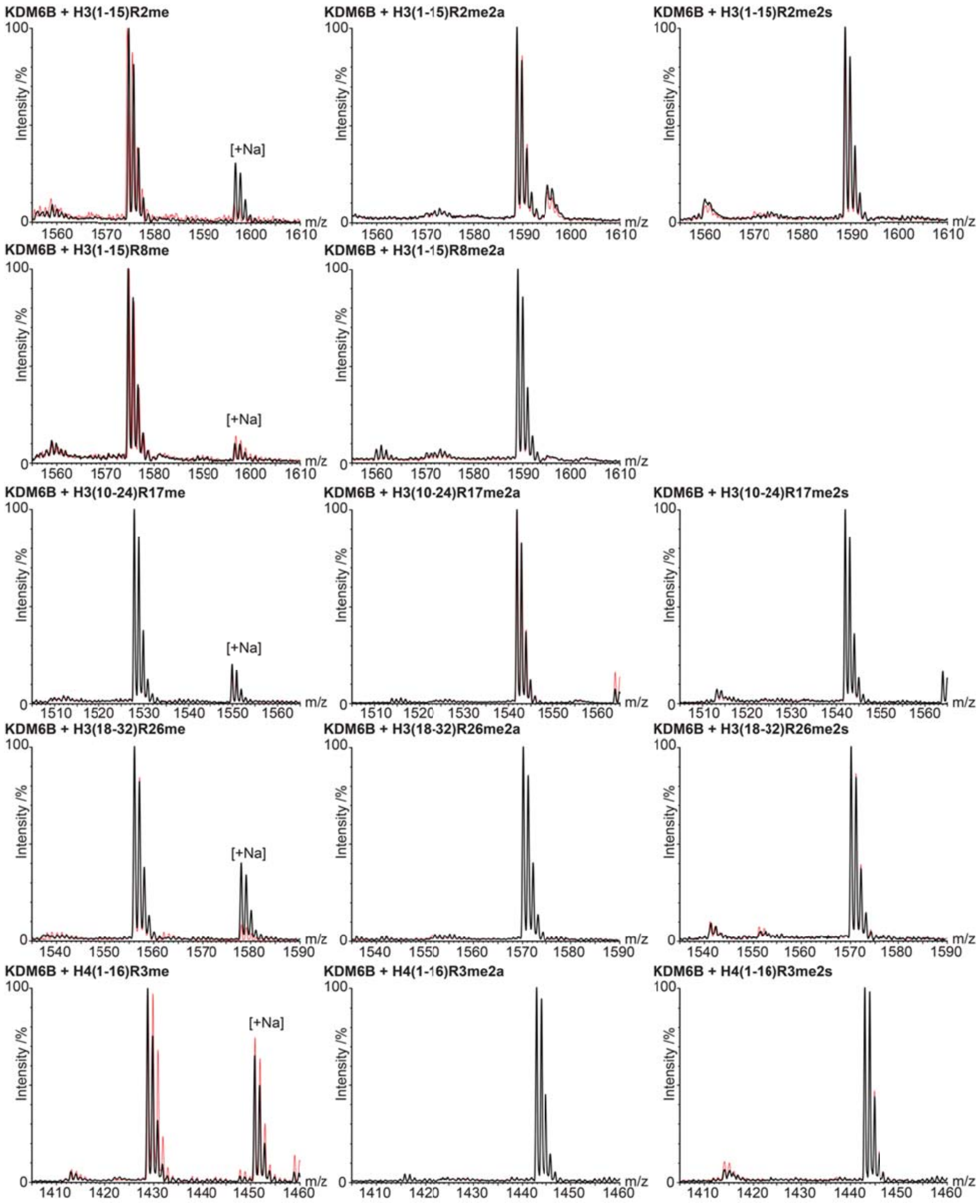

Supplementary Figure 11 KDM6B does not catalyse histone arginine demethylation. MALDI-TOF MS of demethylation of the shown arginine methylated histone peptides catalysed by recombinant KDM6B. Reactions containing enzyme are in red with peptide only controls in black.

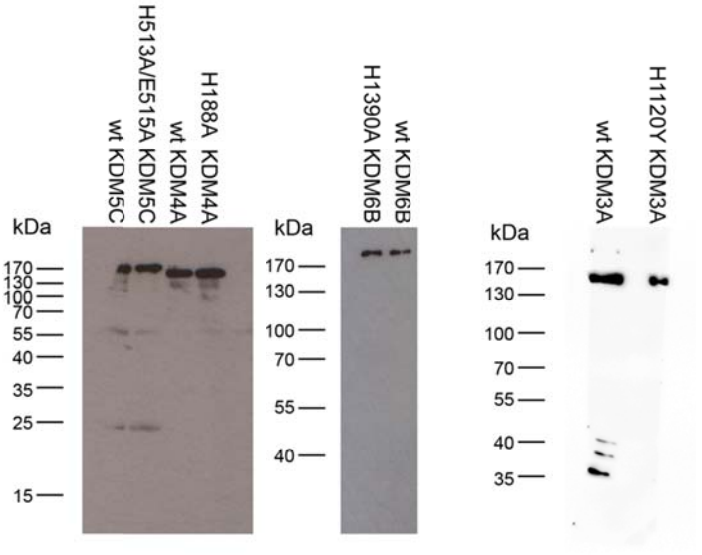

Supplementary Figure 12 Overexpression of full length KDMs in HEK293T cells. Western blot analysis of HEK293T cell lysates exogenously expressing full length Flag-tagged KDMs. KDMs were immunoprecipitated using anti-flag beads to produce full length KDMs for use in on-bead demethylation assays (Fig. 5, Supplementary Fig. 13-16). Full length proteins were detected using anti-Flag primary antibody (for source see Methods).

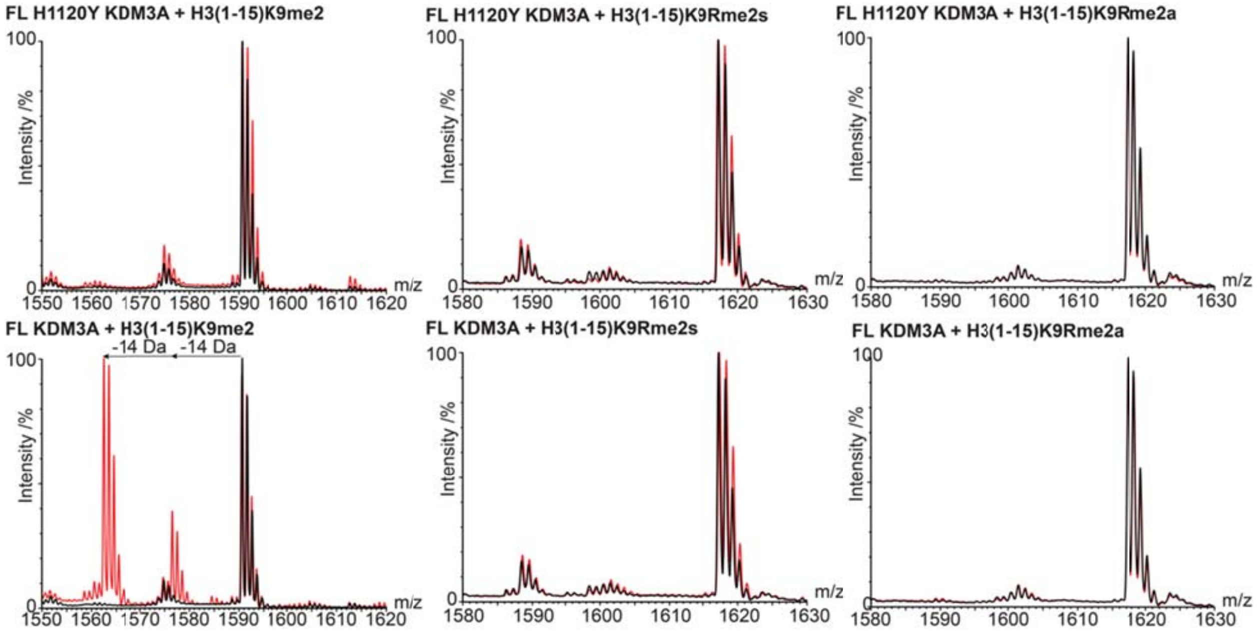

Supplementary Figure 13 Full length KDM3A catalyses arginine and lysine demethylation. MALDI-TOF MS of demethylation of the shown histone peptides as catalysed by full length KDM3A immunoprecipitated from HEK293T cells. Reactions containing enzyme are in red with peptide only controls in black. See main text for other assays (Fig. 5).

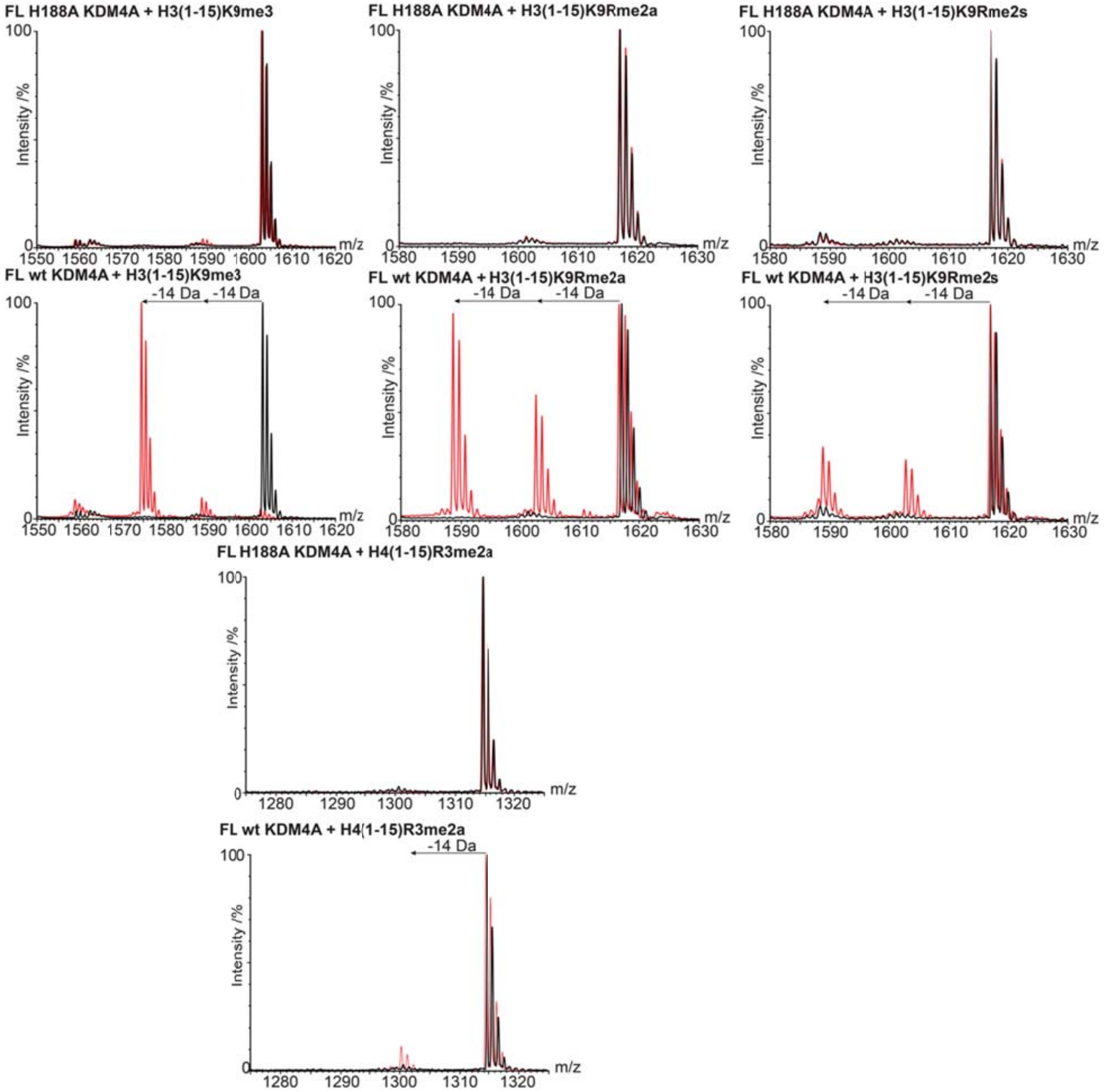

Supplementary Figure 14 Full length KDM4A catalyses arginine and lysine demethylation. MALDI-TOF MS of demethylation of the shown histone peptides as catalysed by full length KDM4A immunoprecipitated from HEK293T cells. Reactions containing enzyme are in red with peptide only controls in black. See main text for other assays (Fig. 5).

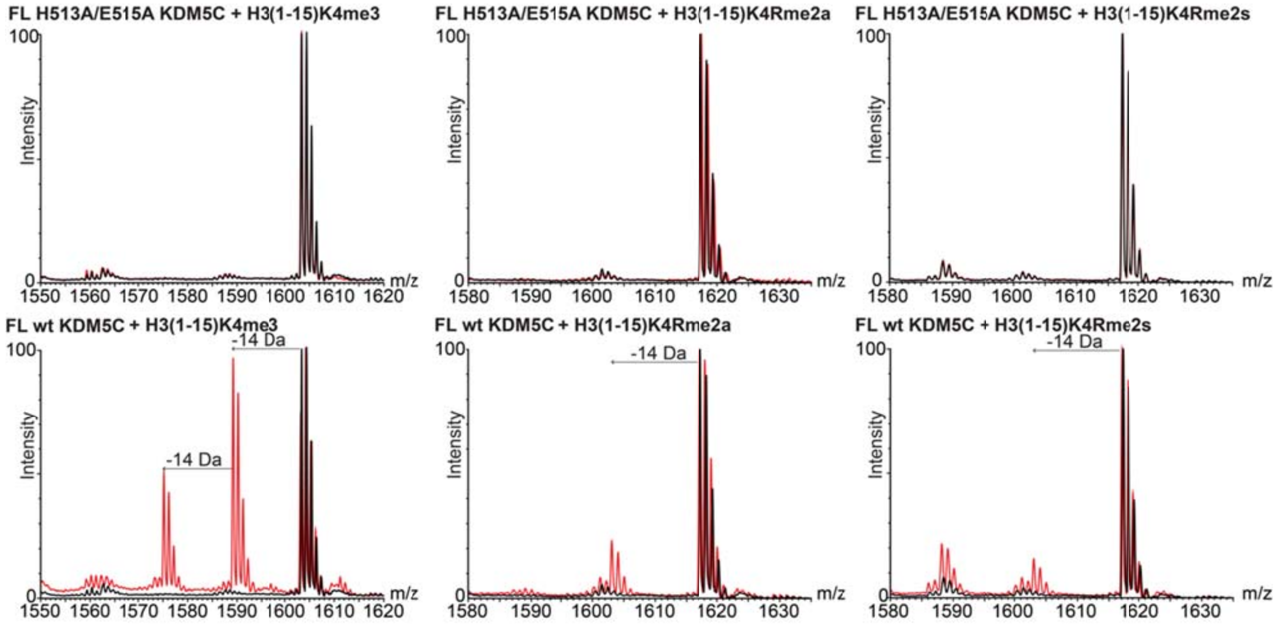

Supplementary Figure 15 Full length KDM5C catalyses arginine and lysine demethylation. MALDI-TOF MS of demethylation of the shown histone peptides as catalysed by full length KDM5C immunoprecipitated from HEK293T cells. Reactions containing enzyme are in red with peptide only controls in black. See main text for other assays (Fig. 5).

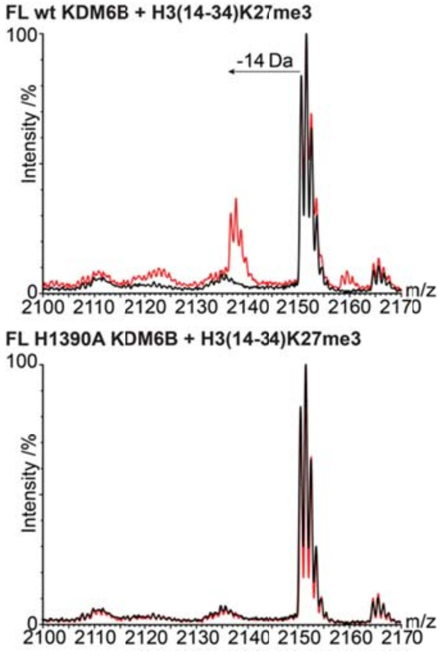

Supplementary Figure 16 Full length KDM6B catalyses arginine and lysine demethylation. MALDI-TOF MS of demethylation of the shown histone peptides as catalysed by full length KDM6B immunoprecipitated from HEK293T cells. Reactions containing enzyme are in red with peptide only controls in black. See main text for other assays (Fig. 5).

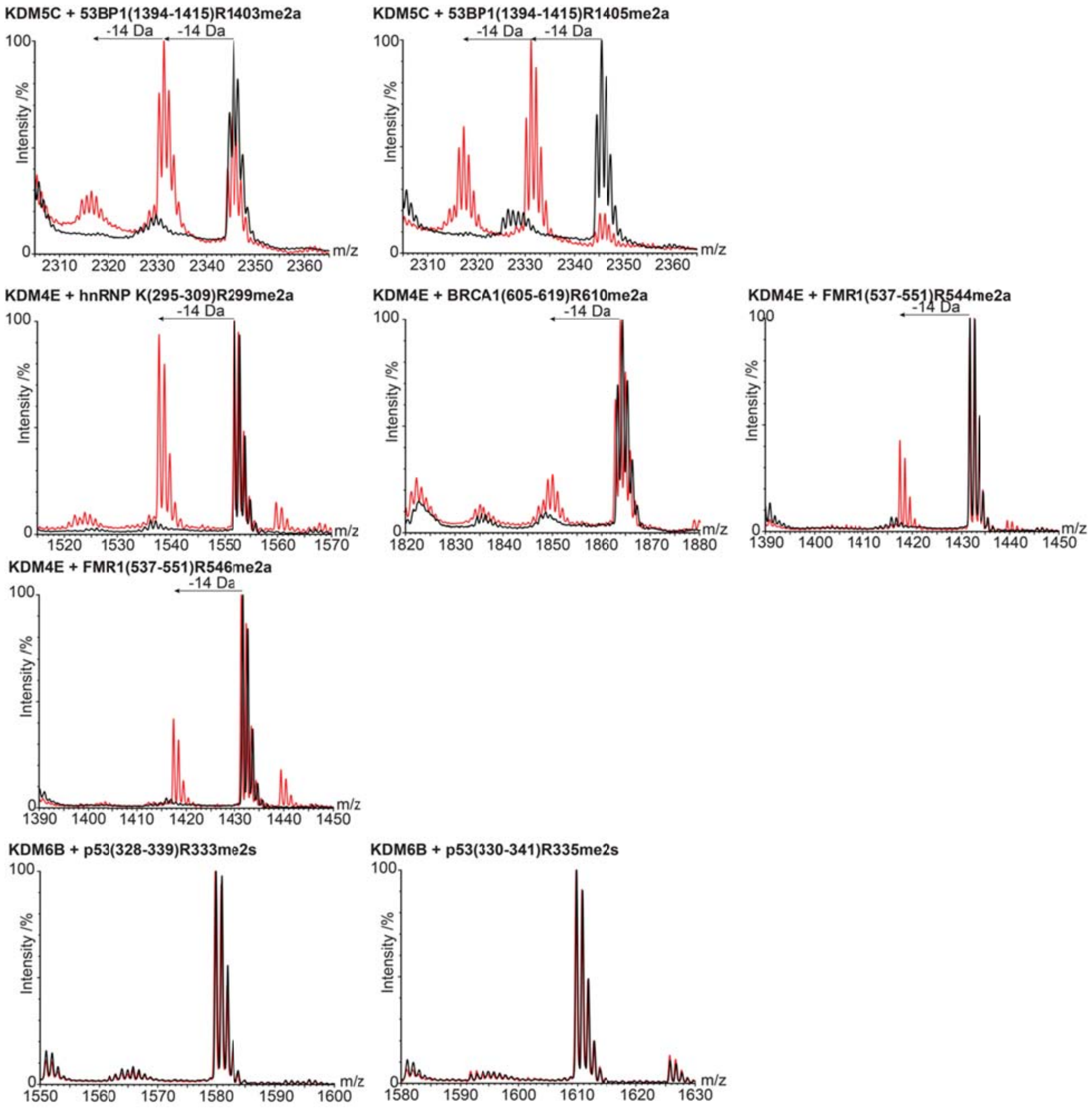

Supplementary Figure 17 KDMs catalyse arginine demethylation in non-histone peptides. MALDI-TOF MS of demethylation of the shown non-histone peptides as catalysed by recombinant KDM5C, KDM4E, and KDM6B. Reactions containing enzyme are in red with peptide only controls in black.

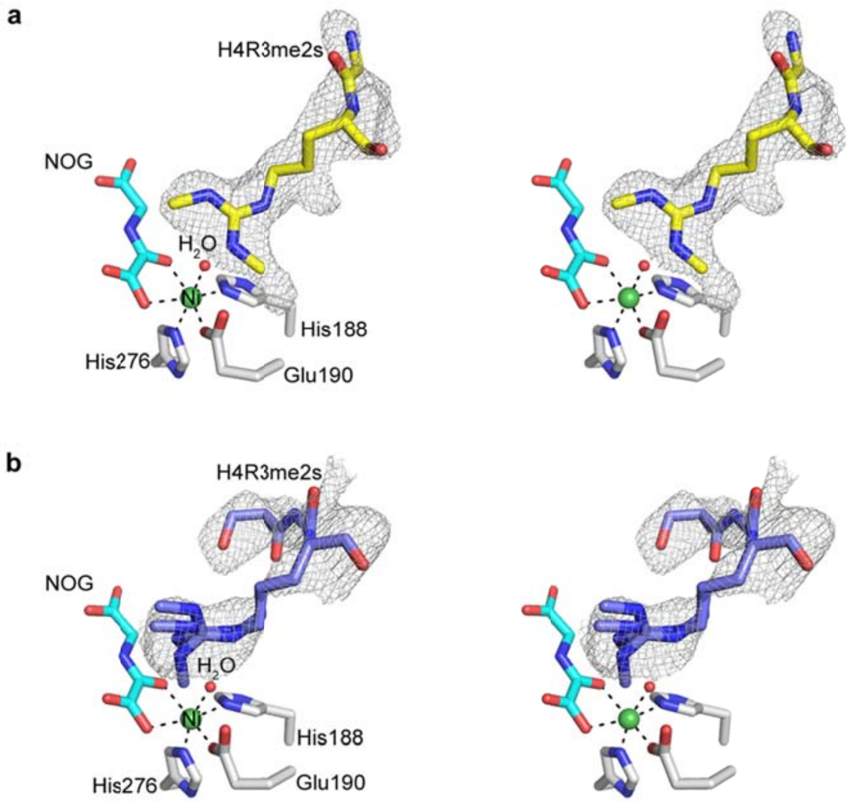

Supplementary Figure 18 Stereoview from a KDM4A.Ni.NOG.H4R3me2s crystal structure. Stereoview from PDB 5FWE showing the *F*o-*F*c OMIT map contoured to 3σ around H4R3me2s peptide residues**.** KDM4A.Ni.NOG.H4R3me2s complex crystallised with two molecules of per asymmetric unit KDM4A (chains A and B). In both KDM4A chains A and B, only weak difference electron density was observed for residues in the H4R3me2s peptides (chains C and D) indicating lower occupancy (<1.00) for substrate residues. This unweighted F_o-F_c OMIT map suggested binding of more than one conformations of the methylated arginine sidechains in KDM4A chains A and B; only the major conformations were modelled and refined in the final structure. In KDM4A Chain A, the methylated arginine conformation is likely catalytically unproductive since both N-methyl groups are too far from the metal (>6.0 Å) **(a)** In chain B, two conformations were refined in both of which one of the *N*-methyl groups was close enough to the metal to enable catalysis (within 4.2 Å) **(b)** as observed for *N*-methyl lysine substrates[1, 2, 3]. Compared to methylated-lysine structures, it is of interest that the *N*-methylated arginine side chain was observed to adopt more than one different extended conformations, which may account for their catalytic differences (Fig 6).

Supplementary Table 1 Peptide sequences used in the biochemical assays. All peptides were prepared as C-terminal amides. In some cases small shifts from these masses are observed in the MS due to shifts in the calibration. In all cases demethylation is observed as a shift of 14 Da from the substrate peak.

**Histone Mark**	**Amino Acid Sequence**	**Monoisotopic Mass / Da**	**Enzyme for which activity is characterised**
H3K4me3	ART-**Kme3**-QTARKSTGGKA	1602	KDM5C
H3K4Rme2a	ART-**Rme2a**-QTARKSTGGKA	1615	
H3K4Rme2s	ART-**Rme2s**-QTARKSTGGKA	1615	
H3K4Rme	ART-**Rme**-QTARKSTGGKA	1601	
H3K9me3	ARTKQTAR-**Kme3**-STGGKA	1602	KDM4A/E
H3K9me2	ARTKQTAR-**Kme2**-STGGKA	1587	KDM3A
H3K9Rme2a	ARTKQTAR-**Rme2a**-STGGKA	1615	
H3K9Rme2s	ARTKQTAR-**Rme2s**-STGGKA	1615	
H3K9Rme	ARTKQTAR-**Rme**-STGGKA	1601	
H3K4me3K9me2	ART-Kme3-QTAR-**Kme2**-STGGKA	1631	KDM7B
H3K4me3K9Rme2a	ART-Kme3-QTAR-**Rme2a**-STGGKA	1658	
H3K4me3K9Rme2s	ART-Kme3-QTAR-**Rme2s**-STGGKA	1658	
H3K4me3K9Rme	ART-Kme3-QTAR-**Rme**-STGGKA	1644	
H3K27me3	KQLATKAAR-**Kme3**-SAPSTG	1656	KDM6B
H3K27Rme2a	KQLATKAAR-**Rme2a**-SAPAT	1596	
H3K27Rme2s	KQLATKAAR-**Rme2s**-SAPAT	1596	
H3K27Rme	KQLATKAAR-**Rme**-SAPAT	1582	
H3K27me3 (21mer)	KAPRKQLATKAAR-**Kme3**-SAPATGG	2150	KDM6B
H3K27Rme2a (21mer)	KAPRKQLATKAAR-**Rme2a**-SAPATGG	2163	
H3K36me2	APATGGV-**Kme2**-KPHRYRP	1661	KDM2A
H3K36Rme2a	APATGGV-**Rme2a**-KPHRYRP	1689	
H3K36Rme2s	APATGGV-**Rme2s**-KPHRYRP	1689	
H3K36Rme	APATGGV-**Rme**-KPHRYRP	1675	
H3R2me2a	A-**Rme2a**-TKQTARKSTGGKA	1587	
H3R2me2s	A-**Rme2a**-TKQTARKSTGGKA	1587	
H3R2me	A-**Rme**-TKQTARKSTGGKA	1573	
H3R2Kme3	A-**Kme3**-TKQTARKSTGGKA	1575	
H3R8me2a	ARTKQTA-**Rme2a**-KSTGGKA	1587	
H3R8me2s	ARTKQTA-**Rme2s**-KSTGGKA	1587	
H3R8me	ARTKQTA-**Rme**-KSTGGKA	1573	
H3R17me2a	STGGKAP-**Rme2a**-KQLATKA	1540	
H3R17me2s	STGGKAP-**Rme2a**-KQLATKA	1540	
H3R17me	STGGKAP-**Rme**-KQLATKA	1526	
H3R26me2a	KQLATKAA-**Rme2a**-KSAPAT	1568	
H3R26me2s	KQLATKAA-**Rme2a**-KSAPAT	1568	
H3R26me	KQLATKAA-**Rme**-KSAPAT	1554	
H4R3me2a (15mer)	SG-**Rme2a**-GKGGKGLGKGGA	1314	
H4R3me2s (15mer)	SG-**Rme2s**-GKGGKGLGKGGA	1314	
H4R3me2a (16mer)	SG-**Rme2a**-GKGGKGLGKGGAK	1441	
H4R3me2s (16mer)	SG-**Rme2s**-GKGGKGLGKGGAK	1441	
H4R3me	SG-**Rme**-GKGGKGLGKGGAK	1427	
53BP1(1394-1415) R1401me2a	GKAPVTP-**Rme2a**-GRGRRGRPPSRTTG	2345	
53BP1(1394-1415) R1403me2a	GKAPVTPRG-**Rme2a**-GRRGRPPSRTTG	2345	

53BP1(1394-1415) R1405me2a	GKAPVTPRGRG-**Rme2a**-RGRPPSRTTG	2345	
BRCA1(605-619)R610me2a	APKKN-**Rme2a**-LRRKSSTRH	1862	
hnRNPK(295-309)R296me2a	G-**Rme2a**-GGRGGSRARNLPL	1550	
hnRNPK(295-309)R299me2a	GRGG-**Rme2a**-GGSRARNLPL	1550	
FMR1(537-551)R544me2a	GGRGQGG-**Rme2a**-GRGGGFK	1430	
FMR1(537-551)R546me2a	GGRGQGGRG-**Rme2a**-GGGFK	1430	
p53(332-343)R333me2s	IRGRE-**Rme2s**-FEMFRE	1652	
p53(328-339)R335me2s	FTLQI-**Rme2s**-GRERFE	1578	
p53(330-341)R336me2s	LQIRG-**Rme2s**-ERFEMF	1608	

Supplementary Table 2 Assay conditions with truncated recombinant protein.

**Enzyme**	**[2OG] / μM**	**[Ascorbate] / μM**	**[Fe^{II}] / μM**	**Buffer Conditions**
KDM2A	100	100	10	50 mM HEPES pH 7.5
KDM3A	100	100	50	50 mM HEPES pH 7.5
KDM4E	200	100	10	50 mM HEPES pH 7.5
KDM5C	100	100	10	50 mM HEPES pH 7.5
KDM6B	100	100	10	50 mM HEPES pH 7.5, 150 mM NaCl
PHF8	100	100	10	100 mM HEPES pH 7.5, 500 mM NaCl

Supplementary Table 3 Assay conditions with full length Flag-tagged KDMs.

**Enzyme**	**[2OG] / mM**	**[Ascorbate] / mM**	**[Fe^{II}] / μM**	**Buffer Conditions**
KDM3A	1	1	50	50 mM HEPES pH 7.5
KDM4A	1	1	50	50 mM HEPES pH 7.5
KDM5C	1	1	50	50 mM HEPES pH 7.5
KDM6B	1	1	50	50 mM HEPES pH 7.5, 150 mM NaCl

Supplementary Table 4. Crystallographic data processing and refinement statistics

**PDB acquisition code**	5FWE
**Data Collection**	
Beamline (Wavelength, Å)	I04-1 (0.91741)
Detector	Pilatus 2M
Data processing	HKL2000[4]
Space Group	$P2_12_12$
Cell dimensions a,b,c (Å)	100.739
	149.400
	57.372
No. of molecules/ ASU	2
Resolution (Å)	49.85 – 2.05 (2.12 – 2.05)*
Completeness (%)	99.7 (99.8)*
Redundancy	7.9 (8.1)*
R_{sym}**	0.1 (1.0)*
Mean I/σ(I)	22.4 (2.5)*
Wilson *B* value ($Å^2$)	33.8
**Refinement**	
R_{work} / R_{free}	0.234 / 0.256
No. reflections	54928 (5334)*
No. atoms[Ψ]	
-Enzyme (A/B)	2881/ 2850
-Metal (A/B)	2/ 2
-Ligand (A/B)	10/ 10
-Peptide (C/D)	17/ 29
-Water	392
B-factors[Ψ]	
-Enzyme (A/B)	47.9/ 47.5
-Metal (A/B)	32.8/ 30.4
-Ligand (A/B)	28.5/ 33.4
-Peptide (C/D)	73.3/ 74.1
-Water	45.9
R.m.s. deviation	
Bond length, Å	0.007
Bond angle, °	1.069

One crystal was used for structure determination.

*Highest resolution shell shown in parenthesis.

[Ψ]Polypeptide chain in parenthesis.

Supplementary References

1 Couture JF, Collazo E, Ortiz-Tello PA, Brunzelle JS, Trievel RC. Specificity and mechanism of JMJD2A, a trimethyllysine-specific histone demethylase. *Nature Structural and Molecular Biology* **14**, 689-695 (2007).

2 Chen Z, *et al.* Structural basis of the recognition of a methylated histone tail by JMJD2A. *Proc Natl Acad Sci U S A* **104**, 10818-10823 (2007).

3 Ng SS*, et al.* Crystal structures of histone demethylase JMJD2A reveal basis for substrate specificity. *Nature* **448**, 87-91 (2007).

4 Otwinowski, Z., Minor, W. Processing of X-ray Diffraction Data Collected in Oscillation Mode. *Methods in Enzymology* **276**, 307-326 (1997).
